# A Superstrong and Ultratough Stretchable Electronic Conductor With Ultradurable Strain‐Insensitive Electromechanical Performance

**DOI:** 10.1002/advs.77457

**Published:** 2026-09-27

**Authors:** Yuxing Shan, Dong Lei, Chengzhi Huang, Chunhong Gong, Jingwei Zhang, Xiaokong Liu

**Affiliations:** ^1^ State Key Laboratory of Supramolecular Structure and Materials College of Chemistry Jilin University Changchun China; ^2^ College of Chemistry and Molecular Sciences Henan University Kaifeng China; ^3^ National & Local Joint Engineering Research Center for Applied Technology of Hybrid Nanomaterials Henan University Kaifeng China

**Keywords:** electromechanical performance, liquid metal, soft electronics, stretchable electronic conductors, supramolecular elastomers

## Abstract

Developing high‐performance stretchable electronic conductors (SECs) is vital for advancing soft electronics and robotics. However, existing SECs still suffer from limited mechanical robustness and vulnerable conductive pathways, undermining their electromechanical stability and durability in stretchable electronic applications. Here, we report a superstrong and ultratough SEC that exhibits ultradurable strain‐insensitive electromechanical performance, deliberately engineered by dispersing a viscoplastic quasi‐solid conductive filler into a superstrong, ultratough supramolecular elastomer. Instead of utilizing liquid metal (LM) as the conductive filler, we developed an LM‐Ag alloy that becomes a viscoplastic quasi‐solid conductor yet exhibits thixotropic flow, while also demonstrating lowered surface tension and enhanced interfacial interaction with the supramolecular elastomer. Our design effectively prevents LM leakage, addressing the formidable challenge encountered in conventional LM‐based SECs. Importantly, the LM‐Ag alloy enables thixotropic flow to establish additional conductive pathways upon stretching, endowing the SEC with strain‐insensitive conductance. The SEC displays superhigh strength (∼20.0 MPa) and ultrahigh toughness (∼66 MJ m^−3^), showing negligible resistance change (*R*/*R*_0_ ≈ 1.07) at 300% strain and <3% resistance increase even after 20 000 stretching‐releasing cycles. Consequently, the SEC affords high‐fidelity electrical signal transmission under stretching, enabling the construction of wearable physiological monitoring and human‐machine interaction systems that maintain functional stability during body movements.

## Graphical abstract

We report a superstrong, ultratough, and fatigue‐resistant stretchable electronic conductor (SEC) integrating a viscoplastic quasi‐solid LM‐Ag alloy with a supramolecular elastomer. Unlike conventional LM, the alloy exhibits thixotropic flow, reduced surface tension, and enhanced interfacial interactions with the elastomer. These characteristics prevent LM leakage and achieve strain‐insensitive conductance. Combined with exceptional mechanical robustness, the SEC displays excellent electromechanical cycling stability.

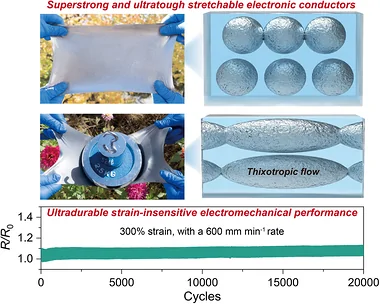

## 1 Introduction

Stretchable electronic conductors (SECs) are vital components for the advancement of next‐generation soft electronics and robotics [1, 2, 3, 4]. An ideal SEC is expected to possess high strength and toughness to withstand repeated stretching, low stiffness to comply with the deformation of target substrates/devices, as well as stable and durable electromechanical performance that ensures low strain sensitivity under prolonged cyclic stretching. However, achieving this combination of mechanical and electrical merits in SECs remains a long‐standing challenge. First, low stiffness and high strength are generally conflicting, as the soft, flexible structures required for low stiffness typically compromise mechanical strength [5, 6, 7, 8, 9, 10, 11]. Second, minimizing strain sensitivity is difficult, since stretching would alter geometrical dimensions and disrupt conductive pathways, thereby increasing electrical resistance [11, 12, 13, 14, 15, 16, 17, 18]. Moreover, achieving electromechanical durability is problematic, as cyclic stretching would induce microstructural damage, degrade conductive pathways, and cause mechanical fatigue, ultimately resulting in the gradual loss of electrical performance [19]. These intrinsically coupled challenges continue to hinder the development of high‐performance SECs.

Embedding rigid conductive fillers (e.g., carbon black, carbon nanotubes, metal particles, metal nanowires) into elastomers is a common strategy for fabricating SECs [20, 21, 22, 23, 24, 25, 26, 27]. However, achieving high conductivity requires a high loading of rigid fillers to establish effective conductive pathways, which inevitably results in high stiffness of the resulting SECs. Moreover, such SECs often exhibit high strain sensitivity, as stretching reduces the conductive filler overlap and disrupts the conductive pathway [28]. To overcome these limitations, dispersing liquid metal (LM) into elastomers (e.g., SBS, SEBS, PDMS) has emerged as a promising alternative [29, 30, 31, 32]. Leveraging the metallic conductivity and inherent fluidity of LM, this approach enables the formation and dynamic reconfiguration of conductive pathways without increasing the stiffness of the resulting SECs [33, 34, 35, 36, 37, 38, 39, 40]. As a result, LM‐based SECs generally exhibit high mechanical compliance and low strain sensitivity. However, LM leakage poses a major challenge for LM‐based SECs, arising from the high fluidity and high surface tension of LM as well as its weak interfacial interaction with the elastomer matrices [13, 41, 42]. The LM leakage severely compromises the electromechanical stability and durability of LM‐based SECs, leading to a continuous increase of strain sensitivity and deterioration of electrical performance upon prolonged cyclic stretching. Moreover, the mechanical robustness of existing LM‐based SECs remains unsatisfactory (tensile strength <5 MPa) for harsh and prolonged mechanical loading, as incorporating LM into elastomers with mediocre mechanical strength further weakens the overall performance [43, 44, 45, 46, 47, 48, 49, 50]. Taken together, developing highly compliant SECs that can simultaneously offer high mechanical robustness, low strain sensitivity, and long‐term electromechanical stability remains a formidable challenge.

**FIGURE 1:**
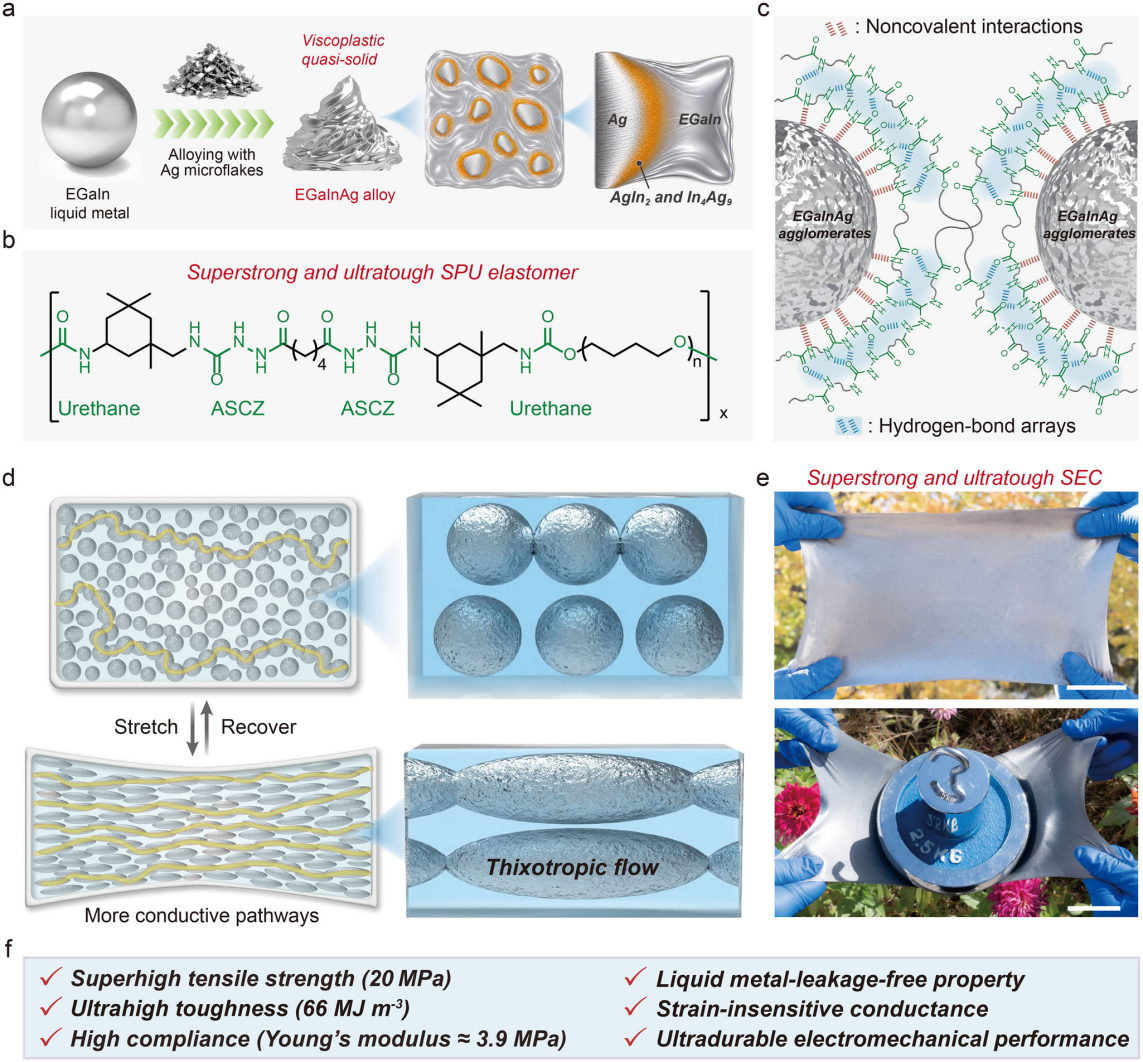
(a) Schematic illustration of the formation of the viscoplastic quasi‐solid EGaInAg alloy by alloying Ag microflakes with EGaIn liquid metal. (b) Molecular structure of the SPU elastomer. (c) Schematic illustration of interfacial interactions between the SPU and EGaInAg agglomerates. (d) Schematic illustration of EGaInAg agglomerates dynamically coalescing and interconnecting under strain. (e) Photographs of a large‐area EGaInAg‐SPU SEC (25 cm × 20 cm × 0.02 cm, top) and its ability to support a 4.0 kg weight (bottom). Scale bars, 5 cm. (f) Summary of the outstanding properties of the EGaInAg‐SPU SEC.

Here, we report a superstrong, ultratough, and highly compliant SEC that delivers ultradurable strain‐insensitive electromechanical performance, achieved by dispersing a viscoplastic quasi‐solid LM‐Ag alloy into a superstrong, ultratough supramolecular poly(urethane‐urea) (SPU) elastomer. In stark contrast to the eutectic gallium‐indium (EGaIn) LM with high fluidity, the EGaInAg alloy, synthesized by alloying Ag microflakes with indium in EGaIn LM, becomes a viscoplastic quasi‐solid conductor with enhanced conductivity and lowered surface tension, while allowing thixotropic flow (Figure 1a). Meanwhile, distinct from the conventional elastomer matrices (e.g., SBS, SEBS, PDMS) that have weak interfacial interactions with LM, the SPU elastomer, rich in acylsemicarbazide (ASCZ) and urethane groups (Figure 1b), establishes abundant and adaptive noncovalent interactions with the EGaInAg alloy via metal‐chelation and hydrogen‐bonding mechanisms (Figure 1c) [51]. Consequently, the quasi‐solid nature of the EGaInAg conductor, together with its strong interfacial interactions with the SPU matrix, effectively prevents LM leakage from the SEC. Importantly, the viscoplastic EGaInAg conductor enables stretching‐induced coalescence in the SEC owing to thixotropic flow, generating additional conductive pathways that enhance the intrinsic conductivity and offset the elongation‐induced resistance increase, ultimately endowing the SEC with nearly strain‐insensitive conductance (Figure 1d). Our SEC combines a superhigh tensile strength (∼20 MPa), remarkable stretchability (∼1000%), and ultrahigh toughness (∼66 MJ m^−3^) with high mechanical compliance (Young's modulus, ∼3.9 MPa), while exhibiting negligible resistance change (*R*/*R*_0_, ∼1.07) at 300% strain and <3% resistance increase even after 20 000 stretching‐releasing cycles (Figure 1e,f). We demonstrate that the SEC is capable of transmitting electrical signals under dynamic stretching at high fidelity, enabling the construction of wearable and stretchable physiological monitoring and human‐machine interaction systems that maintain functional stability during body movements.

## 2 Results and Discussion

### 2.1 Synthesis and Characterization of EGaInAg Alloy

**FIGURE 2:**
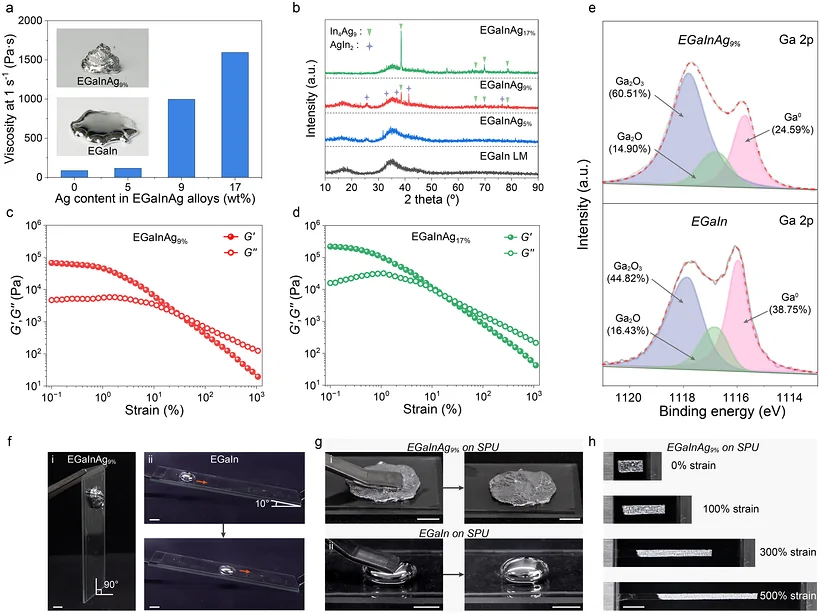
(a) Viscosity of EGaIn LM and EGaInAg alloys measured at a shear rate of 1 s^−^^1^ as a function of Ag weight fraction in the EGaInAg alloys; insets show the viscoplastic quasi‐solid EGaInAg_9%_ alloy (top) and fluid EGaIn LM (bottom). Data were collected at room temperature (25°C). (b) XRD patterns of EGaIn LM and EGaInAg alloys with Ag contents of 5 wt.%, 9 wt.%, and 17 wt.%. Standard reference patterns for AgIn_2_ (PDF#00‐025‐0386) and In_4_Ag_9_ (PDF#00‐029‐0678) are included for comparison. (c,d) Strain‐dependent storage modulus (*G′*) and loss modulus (*G′′*) of EGaInAg_9%_ (c) and EGaInAg_17%_ (d) measured at 1 Hz and 25°C. (e) High‐resolution Ga 2p XPS spectra of EGaInAg_9%_ (top) and EGaIn LM (bottom). f,g) Snapshots of the EGaInAg_9%_ alloy (i) and the EGaIn LM (ii), showing adhesion behavior on the SPU surface (f) and both flattened with a spatula (g), under an Ar atmosphere. Scale bar, 5 mm. (h) Snapshots of EGaInAg_9%_ alloy spread on the SPU film, followed by tensile strains of 0%, 100%, 300%, and 500%. Scale bars, 1 cm.

To address the pervasive LM‐leakage issue yet utilize the flowability of LM for SEC design, we developed a viscoplastic quasi‐solid LM‐Ag alloy and then incorporated it into a superstrong and ultratough SPU elastomer for the fabrication of the target SEC. The LM‐Ag alloys were synthesized by grinding Ag microflakes (5–10 µm in diameter) into the EGaIn LM (75.5 wt.% Ga, 24.5 wt.% In), while the weight fractions of Ag were varied from 5%, 9%, to 17%. The resulting LM‐Ag alloys will be referred to as EGaInAg*_x_*_%_, where *x*% represents the weight fractions of Ag. The reason why we chose Ag microflakes lies in that their flake‐like geometry provides a larger contact interface with EGaIn LM, facilitating In‐Ag alloying during mechanical grinding. In sharp contrast to the EGaIn LM with high fluidity, the EGaInAg alloys become viscoplastic quasi‐solid materials when the Ag weight fraction reaches 9% (inset of Figure 2a and Figures S1–S3). Figure 2a shows that the EGaInAg alloy exhibits a sharp increase in viscosity when the weight fraction of Ag reaches 9%, and the viscosity of the EGaInAg_9%_ alloy (998 Pa·s at a shear rate of 1 s^−1^) is ∼11.5 times higher than that of the EGaIn LM (87 Pa·s at a shear rate of 1 s^−1^, Figure S4). Figure 2b shows X‐ray diffraction (XRD) spectra of the EGaInAg*_x_*_%_ samples, revealing the formation of AgIn_2_ and In_4_Ag_9_ intermetallic compounds when the weight fraction of Ag reaches 9%, thereby confirming the alloying between Ag and In [31, 52, 53, 54, 55]. At the microscopic level, both EGaInAg_9%_ and EGaInAg_17%_ alloys exhibit a solid‐liquid biphasic structure composed of solid Ag microflakes and liquid EGaIn LM. Nevertheless, the in situ formed AgIn_2_ and In_4_Ag_9_ intermetallic compounds improve the interfacial compatibility between Ag microflakes and EGaIn LM, thereby significantly suppressing the free flow of EGaIn LM (Figure 1a). Therefore, the EGaInAg_9%_ and EGaInAg_17%_ alloys exhibit a macroscopically viscoplastic quasi‐solid character while allowing thixotropic flow, which can be quantified by the oscillatory strain‐sweep rheological measurements. Figure 2c,d shows the oscillatory strain‐sweep curves measured at 1.0 Hz and 25°C for EGaInAg_9%_ and EGaInAg_17%_, in which their storage modulus (*G′*) and loss modulus (*G′′*) gradually decrease with increasing strain, followed by a *G′*–*G′′* crossover, indicating a transition from a quasi‐solid state to a viscous‐flow‐dominated state at large strains. Meanwhile, compared to the EGaInAg_9%_ alloy, the EGaInAg_17%_ alloy exhibits much higher *G′* and *G′′* in the quasi‐solid state, indicating that the EGaInAg_17%_ alloy exhibits inferior deformability, which is detrimental for the formation and dynamic reconfiguration of conductive pathways in SECs. Taken together, the viscoplastic quasi‐solid EGaInAg_9%_ alloy with an appropriate modulus represents the optimal conductive filler for the fabrication of our target SECs. Furthermore, Ag incorporation also enhances the electrical conductivity of the EGaInAg alloys (Figure S5), as Ag possesses a markedly higher conductivity (∼6.3 × 10^5^ S cm^−1^) than the EGaIn LM (∼3.4 × 10^4^ S cm^−1^) [1, 56, 57, 58].

Oxidation of EGaIn LM in the EGaInAg alloys plays a significant role in regulating their surface tension, wetting behavior, and interfacial interactions with the SPU elastomer. X‐ray photoelectron spectroscopy (XPS) measurements indicate that the EGaInAg alloys exhibit higher contents of oxidized gallium species (Ga_2_O_3_ and Ga_2_O), compared to the EGaIn LM that was also subjected to similar grinding treatment in air (Figure 2e and Figure S6), as solidification of the EGaInAg alloys suppresses LM flow and prolongs its air exposure during preparation. Consequently, compared to the EGaIn LM, the EGaInAg alloy exhibits lowered surface tension and increased binding sites to SPU (Figure 1c) [59, 60, 61, 62, 63, 64, 65], synergistically enhancing their interfacial compatibility and interactions. Figure 2f_i_,g_i_ show that the EGaInAg_9%_ alloy remains attached to the vertically aligned SPU surface (Movie S1) and can be uniformly spread on SPU, suggesting their strong interfacial interaction. Notably, the boundary of the spread EGaInAg_9%_ alloy (thickness ∼500 µm) follows the stretching of the SPU substrate under tensile strains of 100%, 300%, and 500% (Figure 2h), demonstrating robust interfacial adhesion. In sharp contrast, the EGaIn LM droplet with poor interfacial compatibility with SPU easily rolls off the SPU surface at a tilting angle of 10° (Figure 2f_ii_ and Movie S1), and even when tentatively flattened, it retains a nearly spherical shape and rolls freely across the SPU surface (Figure 2g_ii_). Taken together, we envision that utilizing the viscoplastic quasi‐solid EGaInAg_9%_ alloy with lowered surface tension as the conductive filler can effectively suppress LM leakage from the SECs while retaining sufficient thixotropic flowability for conductive‐pathway reconfiguration.

### 2.2 Fabrication and Structure of EGaInAg‐SPU Composites

We take the superstrong and ultratough SPU elastomer (Figure S7), developed by our group [51], as a mechanically empowering matrix to fabricate SECs through the formation of EGaInAg‐SPU composites. Briefly, the EGaInAg alloy was dispersed into the SPU dimethylacetamide solution under high‐speed homogenization, followed by casting the resulting suspension into a Petri dish (see Experimental Section, Figure S8). After solvent removal under vacuum at elevated temperature, the EGaInAg‐SPU composite was peeled off, yielding a free‐standing SEC denoted as (EGaInAg*_x_*_%_)*_y_*_%_‐SPU, where *y*% represents the weight fraction of the EGaInAg alloy. Note that the peeling process induces percolation of the EGaInAg agglomerates to form conductive pathways, transforming the EGaInAg‐SPU composite into an SEC. To illustrate the critical role of the EGaInAg alloy, we rationally designed two control samples using the EGaIn LM as the conductive filler: (i) EGaIn‐SPU‐Ag, in which the EGaIn LM and Ag microflakes were separately incorporated into SPU at the same relative contents, (ii) EGaIn‐SPU, containing only EGaIn LM without involving Ag microflakes (see the Experimental Section, Figure S9) [29, 30, 31].

**FIGURE 3:**
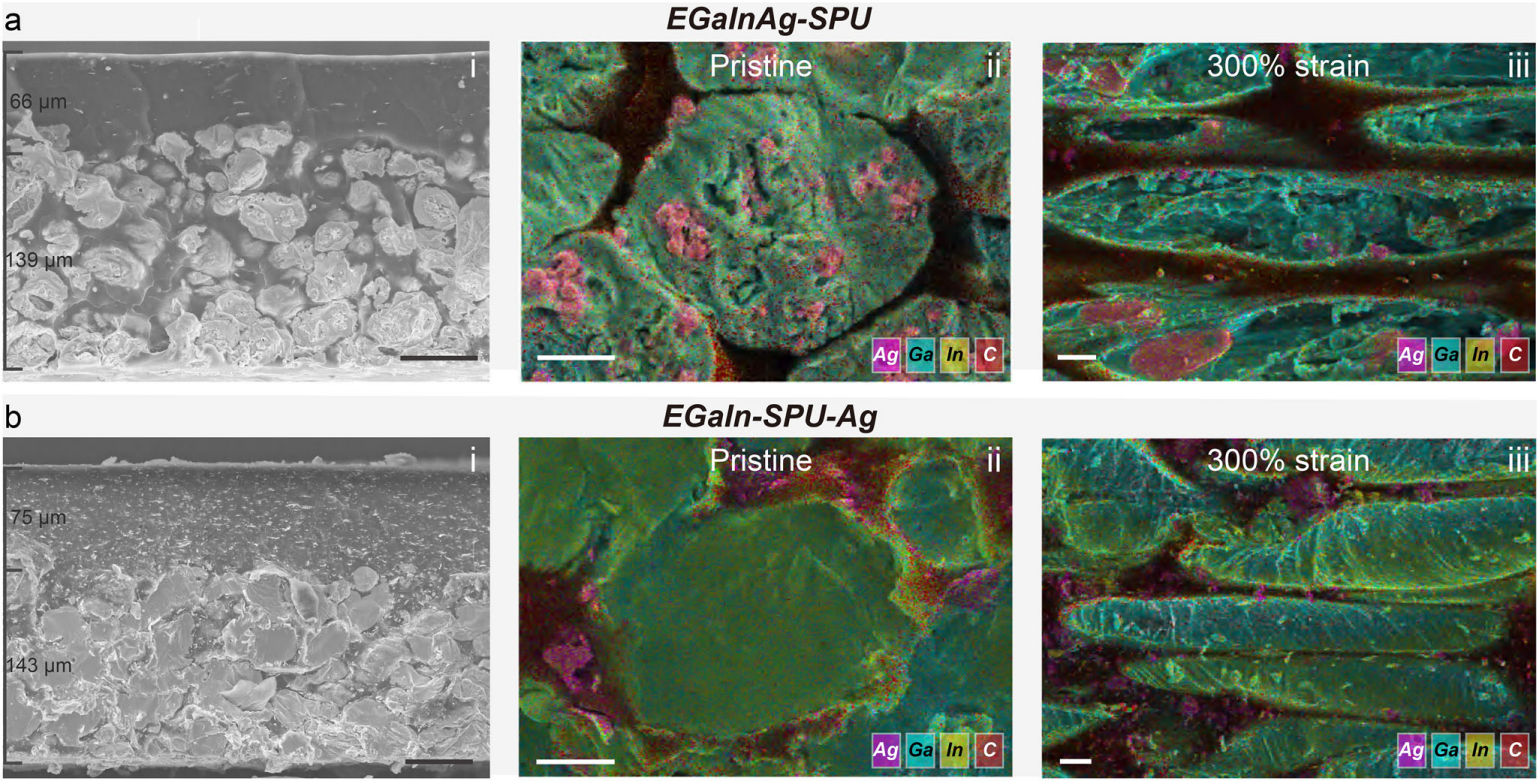
(a, b) Structural characterization of EGaInAg‐SPU (a) and EGaIn‐SPU‐Ag (b): (i) cross‐sectional SEM images; (ii, iii) EDS elemental maps of Ag, Ga, In, and C in the pristine state (ii) and under 300% tensile strain (iii). Scale bars: 50 µm in SEM images and 10 µm in EDS maps.

Taking (EGaInAg_9%_)_85%_‐SPU as an example, its cross‐sectional structure was characterized by scanning electron microscopy (SEM) and compared with that of the EGaIn‐SPU‐Ag sample. Figure 3a_i_,b_i_ show that both samples exhibit a Janus‐like continuous bilayer structure, where the thickness ratio between the metal‐rich and elastomer‐rich layers is about 2:1, and the overall thickness is ∼210 µm. The formation of such a bilayer morphology results from the rapid sedimentation of the EGaInAg_9%_ agglomerates or EGaIn microdroplets during fabrication. In the (EGaInAg_9%_)_85%_‐SPU SEC, the EGaInAg_9%_ agglomerates with diameters of 30–35 µm (Figure S10) display rough surfaces due to the incorporation of Ag microflakes (Figure 3a_i_,a_ii_). Correspondingly, energy‐dispersive X‐ray spectroscopy (EDS) elemental mapping clearly shows co‐localization of In and Ag, confirming the formation of In‐Ag intermetallic compounds due to the alloying effect (Figure 3a_ii_ and Figure S11), consistent with the XRD measurements (Figure S12). Notably, EDS elemental mapping shows that the EGaInAg agglomerates in (EGaInAg_9%_)_85%_‐SPU SEC are elongated along the stretching direction at 300% strain, while the deformed agglomerates retain rough, paste‐like surfaces rather than smooth liquid‐droplet morphology, indicating the viscoplastic quasi‐solid character of the EGaInAg conductive phase (Figure 3a_iii_). Meanwhile, In and Ag remain co‐localized within the deformed EGaInAg agglomerates, indicating that Ag microflakes remain embedded in the EGaIn LM during stretching, thereby maintaining the viscoplastic quasi‐solid character of the EGaInAg conductive phase and suppressing LM leakage. In sharp contrast, the EGaIn‐SPU‐Ag composite comprises EGaIn microdroplets that exhibit smooth surfaces, and the elastomer‐rich phase contains a high concentration of Ag microflakes (Figure 3b_i_,b_ii_). EDS elemental mapping shows that the Ag microflakes are predominantly located in the elastomer matrix without spatial overlap with In (Figure 3b_ii_ and Figure S13), consistent with the XRD patterns that show only characteristic diffraction peaks of Ag (Figure S12). These results indicate that the Ag microflakes are physically isolated from the EGaIn LM in the EGaIn‐SPU‐Ag composite, thereby playing no role in suppressing LM leakage, as similarly observed in previously reported Ag‐nanowire/EGaIn‐based SECs [12, 66, 67]. Figure 3b_iii_ shows EDS elemental mapping of the EGaIn‐SPU‐Ag composite at 300% strain, revealing that Ag microflakes remain physically isolated from the EGaIn microdroplets, while the initially smooth and liquid‐filled EGaIn microdroplets become elongated and oxide‐wrinkled along the tensile direction. The oxide‐wrinkled morphology of the EGaIn microdroplets results from the rupture and reformation of the EGaIn surface oxide layers, providing morphological evidence of LM leakage during mechanical deformation [43]. As a final yet critical point, the Janus‐like architecture of our EGaInAg‐SPU SEC provides a distinct electrical asymmetry with a conductive surface and an opposing insulating layer, which intrinsically facilitates compact and reliable circuit layout in stretchable electronics by suppressing electrical interference with adjacent conductive components or surfaces [68, 69, 70].

### 2.3 Electromechanical Performance

**FIGURE 4:**
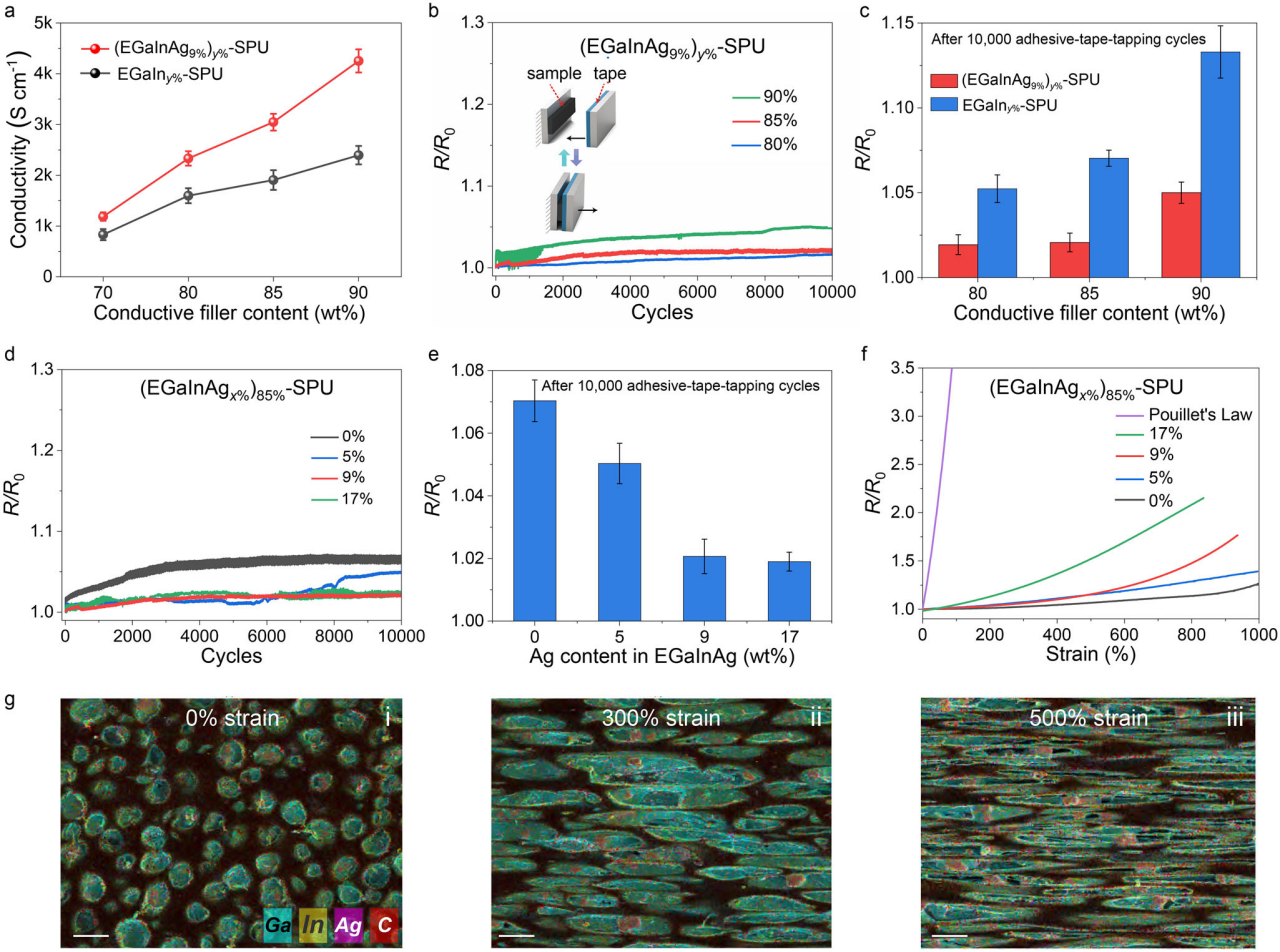
(a) Conductivity of (EGaInAg_9%_)*_y%_*‐SPU and EGaIn*_y%_*‐SPU as a function of conductive filler content. (b–e) Evolution of *R/R*_0_ during cyclic adhesive‐tape‐tapping tests for (EGaInAg_9%_)*_y%_*‐SPU with different filler contents (b) and (EGaInAg*_x_*_%_)_85%_‐SPU with different Ag contents (d), together with the corresponding *R/R*_0_ values after 10 000 cycles (c,e). The inset in b shows the test procedure. (f) *R/R*_0_ of (EGaInAg*_x%_*)_85%_‐SPU as a function of uniaxial strain, and theoretical prediction based on an incompressible bulk conductor [Pouillet's law, *R/R*_0_ = (1 + *ε*)^2^, where *ε* is the applied strain]. (g) EDS elemental maps of the LM‐rich surface of EGaInAg‐SPU at 0%, 300%, and 500% strain. Scale bar, 50 µm.

To attain a high‐performance SEC that simultaneously achieves LM‐leakage‐resistant behavior and strain‐insensitive conductance, we systematically tuned the content and composition of the EGaInAg alloy to fabricate various EGaInAg‐SPU composites. First, EGaInAg_9%_ was selected as the conductive filler, while its content in the EGaInAg_9%_‐SPU composites was varied from 70 wt.% to 90 wt.%. Figure 4a shows that both the EGaInAg_9%_‐SPU composites and EGaIn‐SPU controls exhibit increased conductivity with the increase in filler content. Meanwhile, at a given filler content, the EGaInAg_9%_‐SPU composite possesses a significantly higher conductivity than the Ag‐free EGaIn‐SPU control, owing to the higher intrinsic conductivity of the EGaInAg_9%_ alloy relative to EGaIn LM (Figure S5). To investigate the influence of the filler content on the LM leakage, we performed cyclic adhesive‐tape‐tapping treatments on the samples and monitored their electrical response (Figure S14). Subsequently, the LM leakage can be evaluated by the relative resistance change (*R*/*R*_0_) during the tape‐tapping treatment, defined as the ratio of instantaneous resistance (*R*) to initial resistance (*R*_0_). Figure 4b,c shows that the EGaInAg_9%_‐SPU samples with filler contents of 80 wt.% and 85 wt.% maintain minimal *R*/*R*_0_ values (∼1.02) even after 10 000 tape‐tapping cycles, highlighting their exceptional resistance to LM leakage. By contrast, the EGaInAg_9%_‐SPU with a filler content of 90 wt.% exhibits a markedly higher *R/R*_0_, indicating that excessively high filler loading exceeds the effective confinement capability of the SPU matrix, thereby leading to increased LM leakage. Importantly, all EGaInAg_9%_‐SPU samples exhibit lower *R*/*R*_0_ values as opposed to the Ag‐free EGaIn*_y_*_%_‐SPU controls (Figure 4c and Figure S15), owing to the enhanced LM‐leakage resistance enabled by the viscoplastic quasi‐solid character and lower surface tension of the EGaInAg_9%_ alloy. Collectively, considering the balance of electrical conductivity and LM‐leakage resistance, the EGaInAg filler content was determined to be 85 wt.%.

Next, by fixing the filler content at 85 wt.%, we fabricated three EGaInAg‐SPU composites using various EGaInAg alloys with Ag content (*x*%) varied from 5 wt.%, 9 wt.% to 17 wt.%, and evaluated their LM‐leakage resistance and electrical strain sensitivity. Figure 4d,e shows that the EGaInAg‐SPU samples exhibit decreased *R/R*_0_ values with increasing Ag content during cyclic adhesive‐tape tapping, indicating that higher Ag contents further improve the LM‐leakage resistance of the SECs. Notably, the EGaInAg‐SPU samples containing EGaInAg alloys with Ag contents of 9 wt.% and 17 wt.% maintain minimal *R/R*_0_ values (∼1.02) even after 10 000 tape‐tapping cycles, highlighting their exceptional resistance to LM leakage. To investigate the influence of the Ag content in EGaInAg on the electrical strain sensitivity, the electromechanical responses of the EGaInAg‐SPU SECs were evaluated by monitoring *R/R*_0_ as a function of strain. Figure 4f shows that all the EGaInAg‐SPU SECs exhibit significantly lower resistance‐strain responses than bulk EGaIn LM, which shows *R*/*R*_0_ ≈ 16.0 as predicted by Pouillet's law [71], indicating that conductive pathways in all the EGaInAg‐SPU SECs can reconfigure under strain, albeit to different extents. Notably, the EGaInAg‐SPU samples containing EGaInAg alloys with 17 wt.% Ag show a distinctly higher *R/R*_0_ during stretching than the sample containing the 9 wt.% Ag alloy, indicating higher electrical strain sensitivity because the much higher modulus of EGaInAg_17%_ relative to EGaInAg_9%_ restricts conductive‐pathway reconfiguration during stretching. Taken together, among the various (EGaInAg*_x_*_%_)*_y_*_%_‐SPU composites, (EGaInAg_9%_)_85%_‐SPU demonstrates optimally balanced LM‐leakage resistance and strain‐insensitive conductance, exhibiting a negligible resistance change (*R*/*R*_0_ ≈ 1.07) at 300% strain. To further elucidate the mechanism underlying the strain‐insensitive conductance of (EGaInAg_9%_)_85%_‐SPU, we examined the structural evolution of the internal EGaInAg agglomerates during mechanical stretching via EDS elemental mapping. Figure 4g and Figure S16 show that the EGaInAg agglomerates undergo strain‐induced coalescence and interconnection owing to their thixotropic flow, generating additional conductive pathways that enhance the effective conductivity and offset the elongation‐induced resistance increase, ultimately resulting in strain‐insensitive electrical conductance. Subsequently, (EGaInAg_9%_)_85%_‐SPU will be exclusively studied and referred to as EGaInAg‐SPU, owing to its remarkable LM‐leakage resistance and strain‐insensitive electrical conductance.

### 2.4 Mechanical Robustness and Fatigue Resistance

**FIGURE 5:**
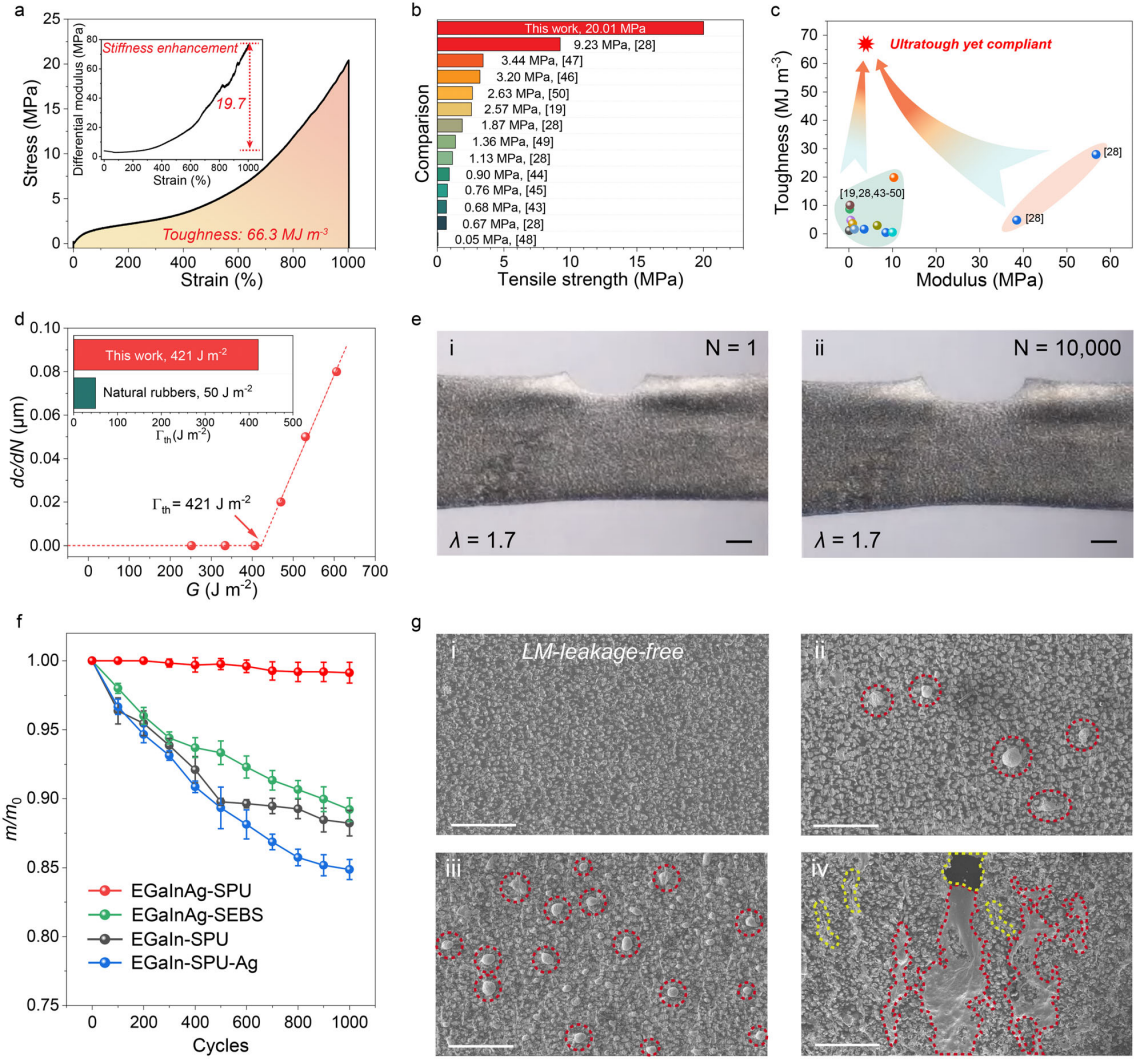
(a) Typical stress‐strain curve of EGaInAg‐SPU. Inset: Differential modulus curve of EGaInAg‐SPU. (b,c) Comparison of tensile strength (b), toughness, and Young's modulus (c) of EGaInAg‐SPU with previously reported LM‐based SECs. Data and references are summarized in Table S1. (d) Crack propagation per loading cycle (*dc/dN*) plotted against energy release rate (*G*). Inset: Comparison of the fatigue threshold between EGaInAg‐SPU and natural rubbers. (e) Photographs of the initial notched specimen (N = 1, λ = 1.7) (i) and the notched specimen after 10 000 cycles (ii). Scale bar, 1 mm. (f,g) Relative mass retention (*m/m*_0_) of SECs after 1000 cycles of tensile loading at 300% strain (f) and SEM images of their surface morphologies (g) for EGaInAg‐SPU (i), EGaIn‐SPU (ii), EGaIn‐SPU‐Ag (iii), and EGaInAg‐SEBS (iv). Scale bar, 500 µm. Red dashed circles highlight leakage of EGaIn LM or EGaInAg, while yellow dashed circles indicate cracks.

To demonstrate the exceptional mechanical robustness and high compliance of our EGaInAg‐SPU SEC, its mechanical performance was evaluated by tensile tests. Figure 5a shows that EGaInAg‐SPU exhibits a superhigh tensile strength of 20.0 ± 0.9 MPa and a remarkable elongation‐at‐break of 1023% ± 43%, resulting in an ultrahigh toughness of 66.3 ± 6.1 MJ m^−3^. Notably, the EGaInAg‐SPU SEC with a conductivity of ∼3.0 × 10^3^ S cm^−1^ achieves a record‐high tensile strength among state‐of‐the‐art LM‐based SECs with adequate electrical conductivity (>1.0 × 10^2^ S cm^−1^, Figure 5b and Table S1) [19, 28, 43, 44, 45, 46, 47, 48, 49, 50]. The outstanding mechanical robustness of EGaInAg‐SPU arises from the intrinsically superrobust SPU matrix with a tensile strength of ∼75.6 MPa and an elongation‐at‐break of ≈1520% [51], while the Janus‐like bilayer structure decouples mechanical properties and electrical conductivity, thereby enabling simultaneous achievement of exceptional mechanical robustness and high conductivity. Despite being superstrong, the EGaInAg‐SPU SEC retains high compliance with a low Young's modulus of ∼3.9 MPa, ascribed to the high content of the viscoplastic EGaInAg conductive filler. Remarkably, EGaInAg‐SPU displays a J‐shaped true stress‐strain curve (Figure S17), indicating its strain‐stiffening behavior with a stiffness enhancement factor as high as 19.7 because of the strain‐induced crystallization of the SPU chains (inset of Figure 5a) [51, 72]. Such a human skin‐like strain‐stiffening behavior imparts outstanding damage‐resistance, which is highly desirable for stretchable electronics [5, 73, 74, 75]. Taken together, our EGaInAg‐SPU SEC surpasses existing state‐of‐the‐art SECs in terms of its distinctive combination of ultrahigh toughness and low modulus (i.e., high compliance, Figure 5c), fulfilling a critical mechanical demand for soft electronics [19, 28, 43, 44, 45, 46, 47, 48, 49, 50].

We further evaluated the fatigue resistance of the EGaInAg‐SPU SEC by measuring its fatigue threshold using pre‐notched samples [76, 77, 78], as fatigue resistance is a key requirement for SECs subjected to long‐term cyclic stretching. Briefly, the pre‐notched EGaInAg‐SPU samples (5 mm width with 1 mm notch) were challenged by cyclic tensile loading at varied stretch ratios (*λ*), followed by establishing the relationship between crack growth per cycle (*dc*/*dN*) and applied energy release rate (*G*) (Figure S18). Linear extrapolation of the *dc/dN*‐*G* curve to its intersection with the abscissa yields a fatigue threshold (*Γ_th_*) of approximately 421 J m^−2^ (Figure 5d), which is over 8‐fold greater than that of the natural rubber (*Γ_th_* ≈ 50 J m^−2^) [76, 79]. This high fatigue threshold implies that EGaInAg‐SPU can endure unlimited cyclic loading without crack growth, as long as the applied energy release rate stays below *Γ_th_* (421 J m^−2^). In particular, the pre‐notched EGaInAg‐SPU sample exhibits no crack propagation even after enduring 10 000 cycles of tensile loading at 70% strain (Figure 5e), demonstrating its robustness for prolonged cyclic operation in stretchable electronics. The high fatigue resistance of EGaInAg‐SPU arises from the synergistic contribution from the SPU matrix and the embedded EGaInAg agglomerates. The SPU matrix features an elastomer network crosslinked via abundant H‐bond arrays formed by aggregation of the acylsemicarbazide and urethane motifs (Figure 1c). Under stress, these hydrogen‐bond arrays undergo dynamic dissociation and reformation, allowing stick‐slip motion of the elastomer chains that mitigates stress concentration at the crack tip [51]. In parallel, the embedded EGaInAg agglomerates elongate along the loading direction, deflecting and blunting crack propagation paths (Figure S19) [80, 81]. Together, these combined molecular and structural effects endow the EGaInAg‐SPU SEC with outstanding fatigue resistance.

### 2.5 Structural and Electromechanical Cycling Stability

Structural stability under long‐term cyclic stretching is essential for SECs to achieve reliable and durable performance in practical stretchable electronics. The structural stability of EGaInAg‐SPU was evaluated by measuring the LM leakage and structural integrity under prolonged cyclic tensile loading. The EGaInAg‐SPU sample was subjected to 1000 cycles of tensile loading at 300% strain with the possibly leaked LM removed using adhesive tape after every 100 cycles, whereupon the relative mass change (*m/m*_0_) was monitored, and the surface morphology was examined by SEM. Figure 5f,g_i_ demonstrate that EGaInAg‐SPU exhibits a negligible mass loss (<1%) without structural cracks or leaked LM observed on its surface after the cyclic loading test, suggesting its LM‐leakage‐free behavior and outstanding structural stability under prolonged cyclic loading (Figures S20 and S21). To elucidate the critical roles of the EGaInAg alloy and the SPU elastomer, three control samples were taken for direct comparison: (i) the Ag‐free EGaIn‐SPU composite, (ii) the EGaIn‐SPU‐Ag composite comprising spatially isolated EGaIn LM and Ag microflakes, and (iii) the EGaInAg‐SEBS composite made of the EGaInAg alloy and commercial SEBS elastomer. In sharp contrast to EGaInAg‐SPU, the Ag‐free EGaIn‐SPU, EGaIn‐SPU‐Ag, and EGaInAg‐SEBS composites exhibit 12%, 15%, and 11% weight losses after the cyclic loading test, respectively. SEM images show that substantial leaked LM droplets are distributed on the EGaIn‐SPU and EGaIn‐SPU‐Ag surfaces, while microcracks are observed on the EGaInAg‐SEBS sample, resulting in the leakage of the EGaInAg alloy that merges into continuous patches (Figure 5g_ii_–g_iv_ and Figure S20). These findings highlight that the viscoplastic quasi‐solid nature of the EGaInAg alloy and the exceptional mechanical robustness of the SPU elastomer, as well as their strong interfacial interactions, synergistically contribute to the long‐term cycling stability of the EGaInAg‐SPU SEC.

**FIGURE 6:**
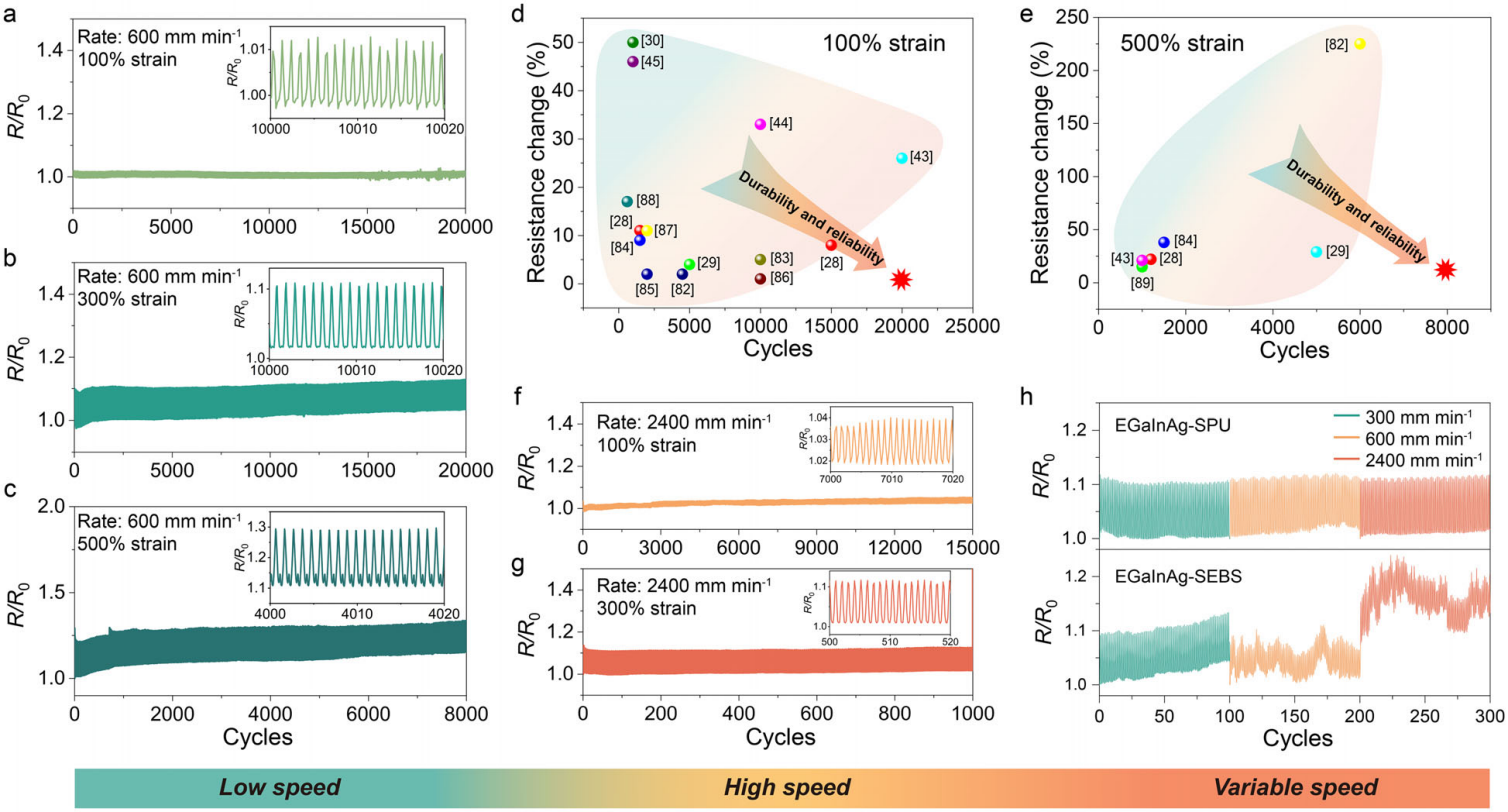
(a–c,f–h) *R*/*R*_0_ of EGaInAg‐SPU under repeated stretching at different strains and stretching speeds: 100% (a), 300% (b), and 500% (c) strain at a stretching speed of 600 mm min^−^^1^; 100% (f) and 300% (g) strain at a stretching speed of 2400 mm min^−^^1^; and 300% strain under variable‐speed stretching at 300, 600, and 2400 mm min^−^^1^ with 100 cycles at each speed (h), with EGaInAg‐SEBS included for comparison. (d,e) Comparison of the electromechanical cycling stability at 100% (d) and 500% (e) strain between the EGaInAg‐SPU and previously reported LM‐based SECs. Data and references are provided in Tables S2 and S3.

Practical applications of SECs in wearable and stretchable electronics demand stable electrical conductance with low strain sensitivity throughout prolonged cyclic stretching. The electromechanical stability and durability of the EGaInAg‐SPU SEC were further tested by monitoring *R*/*R*_0_ under repeated stretching at various strains and strain rates. Figure 6a–c show that, under a strain rate of 600 mm min^−1^, the peak *R*/*R*_0_ values remain at ∼1.01, 1.1, and 1.3 throughout the prolonged cyclic stretching at 100%, 300%, and 500% strain, respectively, indicating nearly strain‐insensitive electrical conductance. Moreover, the resistance (measured at the unstretched state) shows negligible changes (∼1% and ∼3%) even after 20 000 cycles at 100% and 300% strain, and increases to only ∼15% at 500% strain after 8000 cycles, demonstrating excellent electromechanical durability. Compared to the previously reported state‐of‐the‐art LM‐based SECs, EGaInAg‐SPU achieves the lowest resistance change at the highest cycling numbers under both 100% and 500% strain (Figure 6d,e and Tables S2 and S3) [28, 29, 30, 43, 44, 45, 82, 83, 84, 85, 86, 87, 88, 89].

The electromechanical durability of EGaInAg‐SPU was further evaluated at high and variable strain rates. Even under an ultrahigh strain rate of 2400 mm min^−1^, the resistance also exhibits a negligible change at 100% strain (3% increase) after 15 000 cycles and at 300% strain (2% increase) after 1000 cycles (Figure 6f,g). Further, EGaInAg‐SPU was subjected to cyclic stretching at 300% strain under three alternating strain rates of 300, 600, and 2400 mm min^−1^ with 100 cycles applied at each rate. Figure 6h shows that the peak *R*/*R*_0_ values remain stable at ∼1.1, and the resistance increase is also negligible (∼2%) after 300 total cycles. In contrast, EGaInAg‐SEBS shows pronounced fluctuations in peak *R*/*R*_0_ (1.06–1.24) during the cyclic stretching, along with an over sixfold higher resistance increase (∼13%) after 300 cycles. These results demonstrate that EGaInAg‐SPU maintains robust electromechanical stability under both high‐rate and variable‐rate cyclic stretching, owing to the viscoplastic quasi‐solid EGaInAg agglomerates that stabilize conductive pathways and the fatigue‐resistant SPU matrix that preserves structural integrity.

### 2.6 Application Demo of EGaInAg‐SPU in Stretchable Electronic Systems

**FIGURE 7:**
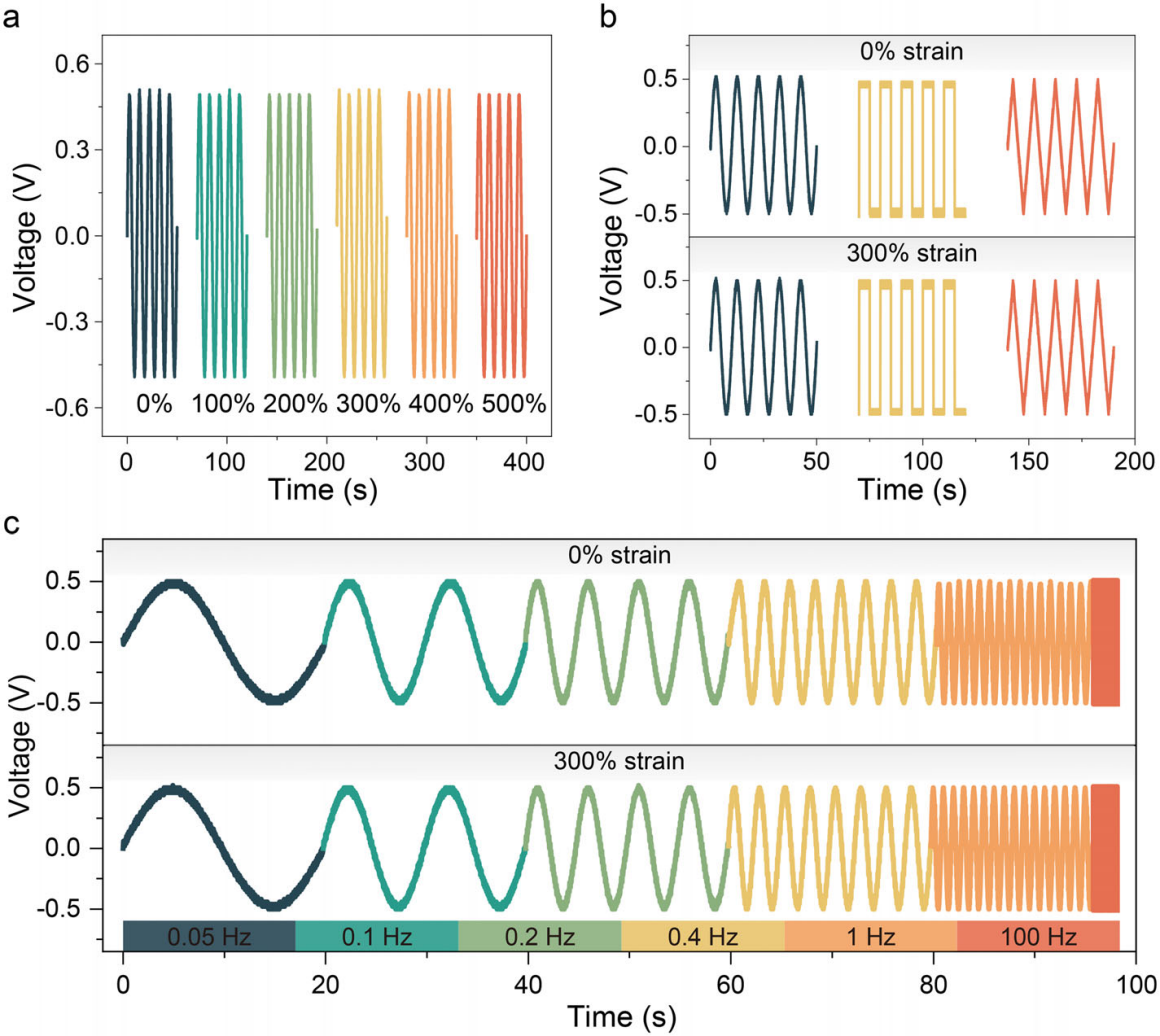
(a) Transmitted alternating voltage signals through the EGaInAg‐SPU SEC under various tensile strains. (b,c) Transmitted alternating voltage signals through the EGaInAg‐SPU SEC with different waveforms (b) and variable frequencies (0.05–100 Hz) (c) under 0% (top) and 300% (bottom) strain.

Stretchable electronics require high‐fidelity signal transmission under dynamic stretching to ensure stable operation [90, 91, 92, 93, 94, 95, 96]. Our EGaInAg‐SPU SEC, exhibiting exceptional mechanical robustness, nearly strain‐insensitive conductance, and ultradurable electromechanical performance, shows great promise for practical applications in stretchable electronics. The EGaInAg‐SPU SEC was subjected to varying tensile strains to assess its signal‐transmission performance during the application of alternating voltage signals with different waveforms and frequencies. Figure 7a and Movie S2 demonstrate that the EGaInAg‐SPU SEC consistently transmits the 0.1 Hz alternating voltage signals at high fidelity under strains up to 500% and during dynamic stretching. Moreover, the EGaInAg‐SPU SEC enables high‐fidelity transmission of the alternating voltage signals with different waveforms and variable frequencies (0.05–100 Hz) under 300% strain (Figure 7b,c), highlighting the reliability of EGaInAg‐SPU for electrical signal transmission under dynamic stretching.

**FIGURE 8:**
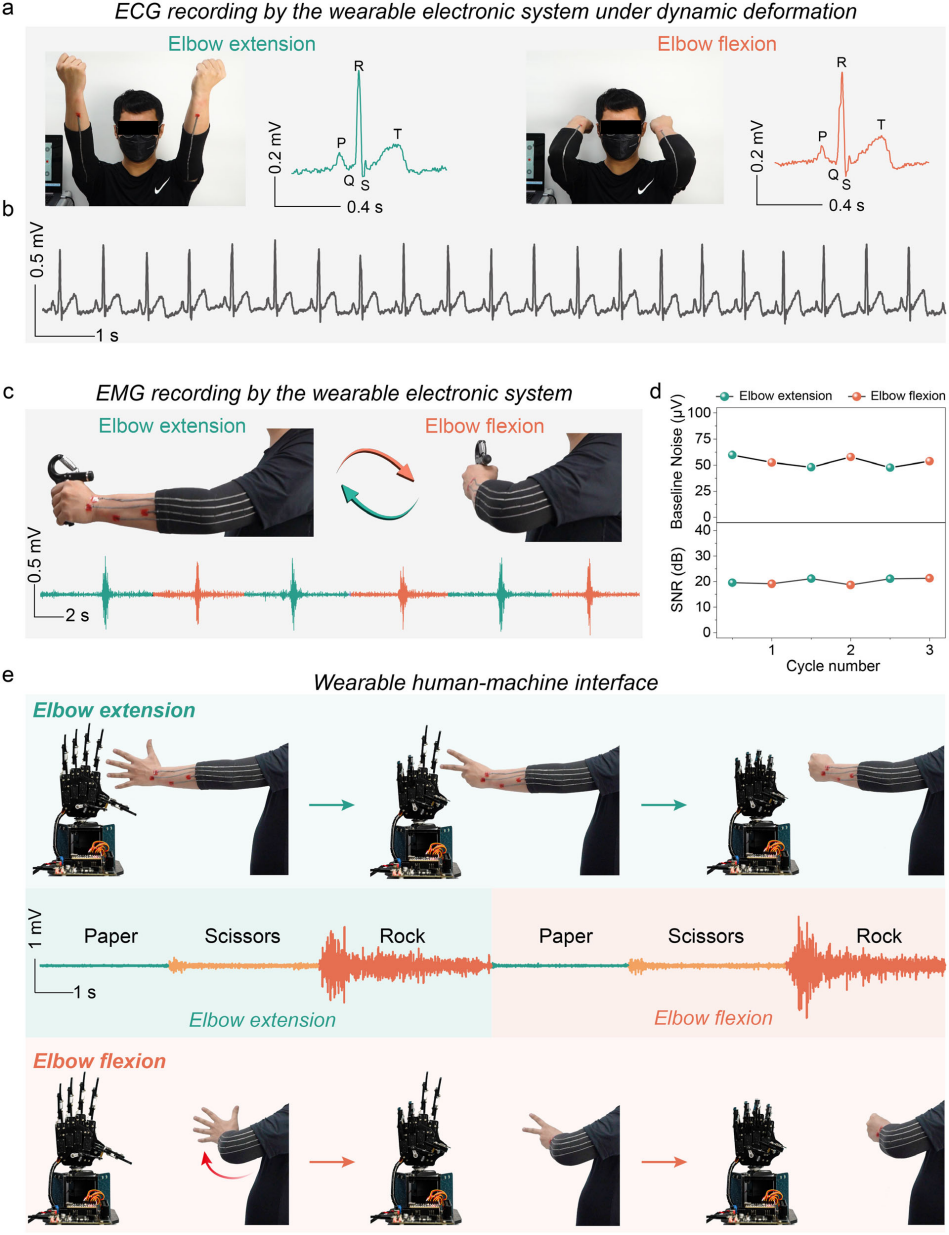
(a,b) Typical ECG signals recorded during elbow extension (a, left), elbow flexion (a, right), and repeated elbow extension and flexion (b). (c,d) EMG signals recorded during grip‐force application under elbow extension and flexion (c), and corresponding baseline noise and signal‐to‐noise ratio (SNR) (d). (e) Snapshots of the wearable human‐machine interface enabling the robotic hand to mimic the volunteer's hand gestures during elbow extension and flexion, with corresponding EMG signals.

We demonstrate a representative application of EGaInAg‐SPU in a wearable physiological monitoring system. The EGaInAg‐SPU SECs were sewn onto an arm sleeve, with one end connected to the on‐skin I‐tattoo electrodes developed by our group and the other end linked to a signal management unit [97], for electrocardiogram (ECG) and electromyogram (EMG) monitoring. Notably, the unique Janus‐like structure of the SEC, featuring a conductive layer and an opposing insulating layer in contact with the skin, effectively eliminates channel crosstalk. As shown in Figure 8a,b and Movie S3, the wearable system maintains high‐fidelity ECG signal recording, with clear P‐waves, QRS complexes, and T‐waves detected during repeated elbow extension and flexion. Figure 8c and Movie S4 show reliable EMG recording by the wearable electronic system during grip‐force application under both elbow extension and flexion. The signals maintain similar baseline noise (51.74 vs. 54.71 µV) and signal‐to‐noise ratio (20.6 vs. 19.8 dB) during elbow extension and flexion states, confirming unaffected signal quality despite the dynamic SEC stretching (Figure 8d). The versatility of the EGaInAg‐SPU SEC was further demonstrated in a wearable human‐machine interaction system. The SECs faithfully transmit EMG signals, which are converted into digital commands for real‐time robotic hand control. As shown in Figure 8e and Movie S5, the robotic hand accurately reproduces the volunteer's gestures during both elbow extension and flexion. The EMG profiles remain nearly identical between the elbow extension and flexion states, indicating that dynamic stretching does not affect signal integrity. These demonstrations underscore the strong promise of the EGaInAg‐SPU SEC for next‐generation stretchable electronics and intelligent wearable systems.

## 3 Conclusion

In summary, we developed a superstrong, ultratough, and fatigue‐resistant SEC by integrating a viscoplastic quasi‐solid EGaInAg alloy with a superstrong, ultratough SPU elastomer, achieving ultradurable strain‐insensitive electromechanical performance. In sharp contrast to the conventional LM, the EGaInAg alloy becomes a viscoplastic quasi‐solid conductor that exhibits thixotropic flow, while also demonstrating increased conductivity, lowered surface tension, and enhanced interfacial interaction with the SPU elastomer. These favorable effects endow the EGaInAg‐SPU SEC with exceptional LM‐leakage resistance, which delivers nearly strain‐insensitive electrical conductance owing to the dynamic coalescence of the viscoplastic EGaInAg conductive filler under stretching. The EGaInAg‐SPU SEC achieves a conductivity of ∼3.0 × 10^3^ S cm^−1^ and exhibits record‐high tensile strength (20.0 ± 0.9 MPa) and toughness (66.3 ± 6.1 MJ m^−3^) among state‐of‐the‐art LM‐based SECs with adequate electrical conductivity (>1.0 × 10^2^ S cm^−1^), while achieving ultradurable strain‐insensitive electromechanical performance with an *R/R*_0_ as low as 1.07 at 300% strain and <3% resistance increase even after 20 000 stretching‐releasing cycles. These mechanical and electromechanical performances establish the EGaInAg‐SPU SEC as a highly reliable and durable signal‐transmitting platform for next‐generation soft electronics, enabling stable operation under intensive mechanical loading and dynamic stretching.

## Author Contributions

**Yuxing Shan**: conceptualization, methodology, software, investigation, formal analysis, visualization, Writing – original draft, Writing – review and editing. **Dong Lei**: data curation, validation. **Chengzhi Huang**: data curation, validation. **Chunhong Gong**: data curation, validation. **Jingwei Zhang**: data curation, validation. **Xiaokong Liu**: conceptualization, methodology, investigation, formal analysis, supervision, resources, project administration, visualization, funding acquisition, writing – original draft, writing – review and editing.

## Conflicts of Interest

The authors declare no conflicts of interest.

## Supporting information




**Supporting File 1**: advs77457‐sup‐0001‐SuppMat.docx.

**Supporting File 2**: advs77457‐sup‐0002‐S1.mp4.

**Supporting File 3**: advs77457‐sup‐0003‐S2.mp4.

**Supporting File 4**: advs77457‐sup‐0004‐S3.mp4.

**Supporting File 5**: advs77457‐sup‐0005‐S4.mp4.

**Supporting File 6**: advs77457‐sup‐0006‐S5.mp4.

## Data Availability

The data that support the findings of this study are available from the corresponding author upon reasonable request.

## References

[1] D. C. Kim , H. J. Shim , W. Lee , J. H. Koo , and D. H. Kim , “Material‐Based Approaches for the Fabrication of Stretchable Electronics,” Advanced Materials 32, no. 15 (2020): 1902743, 10.1002/adma.201902743. 31408223

[2] S. Choi , S. I. Han , D. Kim , T. Hyeon , and D. H. Kim , “High‐Performance Stretchable Conductive Nanocomposites: Materials, Processes, and Device Applications,” Chemical Society Reviews 48, no. 6 (2019): 1566–1595, 10.1039/c8cs00706c. 30519703

[3] N. Matsuhisa , X. Chen , Z. Bao , and T. Someya , “Materials and Structural Designs of Stretchable Conductors,” Chemical Society Reviews 48, no. 11 (2019): 2946–2966, 10.1039/c8cs00814k. 31073551

[4] B. Cheng , J. Zhuo , Y. Zhou , et al., “Design, Fabrication, and Application of Stretchable Electronic Conductors,” Nano‐Micro Letters 18, no. 1 (2026): 166, 10.1007/s40820-025-02009-3. PMC12770152 41489811

[5] J. Wang , B. Wu , P. Wei , S. Sun , and P. Wu , “Fatigue‐Free Artificial Ionic Skin Toughened by Self‐Healable Elastic Nanomesh,” Nature Communications 13, no. 1 (2022): 4411, 10.1038/s41467-022-32140-3. PMC9338060 35906238

[6] S. Chen , L. Sun , X. Zhou , et al., “Mechanically and Biologically Skin‐Like Elastomers for Bio‐Integrated Electronics,” Nature Communications 11, no. 1 (2020): 1107, 10.1038/s41467-020-14446-2. PMC7046662 32107380

[7] Z. Jing , A. Sun , Z. He , et al., “Highly Robust and Conductive Polymer Electrodes for Droplet Energy Harvesting and Printable On‐Skin Electronics,” Advanced Materials 37, no. 34 (2025): 2506511, 10.1002/adma.202506511. 40492856

[8] B. Jiao , W. Wang , Y. Feng , et al., “High‐Strength Liquid Metal Composite–Hydrogel Interfaces Enable Robust Stretchable Electronics,” Nature Communications 17, no. 1 (2026): 5322, 10.1038/s41467-026-71920-z. PMC13272957 41991912

[9] W. Wang , Z. Luo , X. Yu , X. Yin , L. Xiang , and A. Pan , “A Highly Permeable and Three‐Dimensional Integrated Electronic System for Wearable Human–Robot Interaction,” Nano‐Micro Letters 18, no. 1 (2026): 128, 10.1007/s40820-025-01974-z. PMC12759031 41482530

[10] Z. Wang , P. Shi , Y. Li , et al., “Abrasion‐Resistant Wearable Skins Based on Bilayered Solid/Liquid Stretchable Conductors,” Nature Communications 17, no. 1 (2026): 3767, 10.1038/s41467-026-70438-8. PMC13106628 41807426

[11] X. Qu , W. Guo , Z. Zhu , S. Chen , S. Tang , and Y. Lin , “Strain‐Programmable Liquid Metal Fibers for Anti‐Interference Electronic Textiles,” Nature Communications 17, no. 1 (2026): 4955, 10.1038/s41467-026-71342-x. PMC13233833 41946706

[12] X. Wang , S. Zheng , J. Xiong , et al., “Stretch‐Induced Conductivity Enhancement in Highly Conductive and Tough Hydrogels,” Advanced Materials 36, no. 25 (2024): 2313845, 10.1002/adma.202313845. 38452373

[13] R. Ma , L. Jia , Z. Guo , et al., “A General Finite‐Gel Strategy for Highly Concentrated Liquid Metal Inks,” Nature Communications 16, no. 1 (2025): 9085, 10.1038/s41467-025-63433-y. PMC12531317 41102153

[14] Y. Lin , X. Zhou , C. Yang , et al., “In Situ Synthesis of Biocompatible and Functionalizable Core–Shell Conductive Nanocomposites for Stretchable Electronics,” Advanced Materials 38, no. 17 (2026): 20928, 10.1002/adma.202520928. 41746185

[15] P. Wu , S. Jia , J. Li , et al., “A Three‐Dimensional Stretchable Core–Shell Cable For Soft And Hybrid Electronics That Is Patternable, Recyclable And Noise‐Resistant,” Nature Electronics 9, no. 5 (2026): 519–531, 10.1038/s41928-026-01596-2.

[16] X. Liu , Q. Wang , S. Zhou , et al., “Stiffness and Interface Engineered Soft Electronics With Large‐Scale Robust Deformability,” Advanced Materials 36, no. 40 (2024): 2407886, 10.1002/adma.202407886. 39180261

[17] W. Gu , Q. Guo , Y. Zhang , et al., “Activation‐Independent Biphasic Liquid Metal Conductor Enables Multilayer Stretchable Electronics,” Materials Today 88 (2025): 89–98, 10.1016/j.mattod.2025.05.020.

[18] X. Chen , B. Wang , J. Duan , et al., “Compression‐Durable Soft Electronic Circuits Enabled by Embedding Self‐Healing Biphasic Liquid‐Solid Metal Into Microstructured Elastomeric Channels,” Advanced Materials 37, no. 21 (2025): 2420469, 10.1002/adma.202420469. 40072334

[19] X. Li , J. Wang , W. Wang , et al., “A Durable Metalgel Maintaining 3×10^6^ S∙M^‒1^ Conductivity Under 1 000 000 Stretching Cycles,” Advanced Materials 37, no. 20 (2025): 2420628, 10.1002/adma.202420628. 40159807

[20] Y.‐A. Chen , S.‐J. Chen , L.‐Y. Lee , et al., “Fluoro‐Based Organic Small Molecules as Sliding Crosslinkers for Boosting Stretchability and Self‐Healability of Polymers for Hybrid Human‐Motion Sensing and Energy Harvesting,” Nano Energy 117 (2023): 108882, 10.1016/j.nanoen.2023.108882.

[21] F. Zhang , D. Ren , L. Huang , et al., “3D Interconnected Conductive Graphite Nanoplatelet Welded Carbon Nanotube Networks for Stretchable Conductors,” Advanced Functional Materials 31, no. 49 (2021): 2107082, 10.1002/adfm.202107082.

[22] Y. J. Fan , X. Li , S. Y. Kuang , et al., “Highly Robust, Transparent, and Breathable Epidermal Electrode,” ACS Nano 12, no. 9 (2018): 9326–9332, 10.1021/acsnano.8b04245. 30118595

[23] Y. Xu , Z. Ye , G. Zhao , et al., “Phase‐Separated Porous Nanocomposite With Ultralow Percolation Threshold for Wireless Bioelectronics,” Nature Nanotechnology 19, no. 8 (2024): 1158–1167, 10.1038/s41565-024-01658-6. PMC11330368 38684805

[24] S.‐H. Sunwoo , S. I. Han , D. Jung , et al., “Stretchable Low‐Impedance Conductor With Ag–Au–Pt Core–Shell–Shell Nanowires and In Situ Formed Pt Nanoparticles for Wearable and Implantable Device,” ACS Nano 17, no. 8 (2023): 7550–7561, 10.1021/acsnano.2c12659. 37039606

[25] C. Zhang , X. Yin , C. Zhou , et al., “Soft and Strong: Elastic Conductors With Bio‐Inspired Self‐Protection,” Advanced Materials 38, no. 4 (2026): 12471, 10.1002/adma.202512471. PMC12810613 41099142

[26] T. Chen , X. Chen , Z. Lin , et al., “Biodegradable, Stretchable, and Self‐Healing Starch‐Based Hydrogel With Intelligent Multi‐Bond Network Facilitated by MXene Nanosheets for Multifunctional Wearable Electronics,” Advanced Materials 38, no. 2 (2026): 12267, 10.1002/adma.202512267. 40990471

[27] M. Kim , S. Jung , S. Kim , et al., “2D Silver Nanosheet Assembly for an Isotropic, Stretchable, and Highly Conductive Nanomembrane,” Advanced Materials 38, no. 20 (2026): 16002, 10.1002/adma.202516002. 41316824

[28] W. Lee , H. Kim , I. Kang , et al., “Universal Assembly of Liquid Metal Particles in Polymers Enables Elastic Printed Circuit Board,” Science 378, no. 6620 (2022): 637–641, 10.1126/science.abo6631. 36356149

[29] D. H. Lee , T. Lim , J. Pyeon , et al., “Self‐Mixed Biphasic Liquid Metal Composite With Ultra‐High Stretchability and Strain‐Insensitivity for Neuromorphic Circuits,” Advanced Materials 36, no. 16 (2024): 2310956, 10.1002/adma.202310956. 38196140

[30] M. Tavakoli , P. Alhais Lopes , A. Hajalilou , et al., “3R Electronics: Scalable Fabrication of Resilient, Repairable, and Recyclable Soft‐Matter Electronics,” Advanced Materials 34, no. 1 (2022): 2203266, 10.1002/adma.202203266. 35697348

[31] A. Hajalilou , A. F. Silva , P. A. Lopes , E. Parvini , C. Majidi , and M. Tavakoli , “Biphasic Liquid Metal Composites for Sinter‐Free Printed Stretchable Electronics,” Advanced Materials Interfaces 9, no. 5 (2022): 2101913, 10.1002/admi.202101913.

[32] D. H. Ho , C. Hu , L. Li , and M. D. Bartlett , “Soft Electronic Vias and Interconnects Through Rapid Three‐Dimensional Assembly of Liquid Metal Microdroplets,” Nature Electronics 7, no. 11 (2024): 1015–1024, 10.1038/s41928-024-01268-z.

[33] M. H. Malakooti , M. R. Bockstaller , K. Matyjaszewski , and C. Majidi , “Liquid Metal Nanocomposites,” Nanoscale Advances 2, no. 7 (2020): 2668–2677, 10.1039/d0na00148a. PMC9419082 36132412

[34] Y. Chen , Z. Zhang , S. He , and G. Liu , “Engineering Liquid Metal Nanoparticles for Wearable Devices,” ACS Nano 20, no. 7 (2026): 5352–5371, 10.1021/acsnano.5c18099. 41665400

[35] J. Wang , T. Ye , Y. Jiao , et al., “A Metalgel With Liquid Metal Continuum Immobilized in Polymer Network,” Advanced Materials 36, no. 49 (2024): 2409137, 10.1002/adma.202409137. 39449216

[36] J. Ma , Z. Liu , Q. K. Nguyen , and P. Zhang , “Lightweight Soft Conductive Composites Embedded With Liquid Metal Fiber Networks,” Advanced Functional Materials 34, no. 31 (2024): 2308128, 10.1002/adfm.202308128.

[37] Q. Lu , Y. Sun , M. Wu , et al., “Multifunctional Nanocomposite Yield‐Stress Fluids for Printable and Stretchable Electronics,” ACS Nano 18, no. 20 (2024): 13049–13060, 10.1021/acsnano.4c01668. 38723037

[38] Q. Lu , T. Fang , C. Ye , et al., “Highly Conductive Liquid Metal Emulsion Gels for Three‐Dimensionally Printed Stretchable Electronics,” Advanced Science 12, no. 36 (2025): 03449, 10.1002/advs.202503449. PMC12462967 40619609

[39] X. Zhou , Y. Liu , Z. Gao , et al., “Biphasic GaIn Alloy Constructed Stable Percolation Network in Polymer Composites Over Ultrabroad Temperature Region,” Advanced Materials 36, no. 14 (2024): 2310849, 10.1002/adma.202310849. 38185468

[40] J. Chen , D. Yi , Y. Ren , et al., “Porous Elastomer Film With Controlled Liquid‐Metal Distribution for Recyclable Highly Customizable and Stretchable Patterned Electronics,” Advanced Materials 37, no. 37 (2025): 2505839, 10.1002/adma.202505839. 40589264

[41] Y. Lu , D. Yu , H. Dong , et al., “Dynamic Leakage‐Free Liquid Metals,” Advanced Functional Materials 33, no. 11 (2023): 2210961, 10.1002/adfm.202210961.

[42] M. D. Dickey , R. C. Chiechi , R. J. Larsen , E. A. Weiss , D. A. Weitz , and G. M. Whitesides , “Eutectic Gallium‐Indium (EGaIn): A Liquid Metal Alloy for the Formation of Stable Structures in Microchannels at Room Temperature,” Advanced Functional Materials 18, no. 7 (2008): 1097–1104, 10.1002/adfm.200701216.

[43] Z. Ma , Q. Huang , Q. Xu , et al., “Permeable Superelastic Liquid‐Metal Fibre Mat Enables Biocompatible and Monolithic Stretchable Electronics,” Nature Materials 20, no. 6 (2021): 859–868, 10.1038/s41563-020-00902-3. 33603185

[44] F. Chen , Q. Zhuang , Y. Ding , et al., “Wet‐Adaptive Electronic Skin,” Advanced Materials 35, no. 49 (2023): 2305630, 10.1002/adma.202305630. 37566544

[45] R. Tutika , A. B. M. T. Haque , and M. D. Bartlett , “Self‐Healing Liquid Metal Composite for Reconfigurable and Recyclable Soft Electronics,” Communications Materials 2, no. 1 (2021): 64, 10.1038/s43246-021-00169-4.

[46] L. Ai , W. Lin , C. Cao , et al., “Tough Soldering for Stretchable Electronics by Small‐Molecule Modulated Interfacial Assemblies,” Nature Communications 14, no. 1 (2023): 7723, 10.1038/s41467-023-43574-8. PMC10673831 38001116

[47] A. Fassler and C. Majidi , “Liquid‐Phase Metal Inclusions for a Conductive Polymer Composite,” Advanced Materials 27, no. 11 (2015): 1928–1932, 10.1002/adma.201405256. 25652091

[48] W. Zang , Y. Wang , W. Wu , et al., “Superstretchable Liquid‐Metal Electrodes for Dielectric Elastomer Transducers and Flexible Circuits,” ACS Nano 18, no. 1 (2024): 1226–1236, 10.1021/acsnano.3c12210. 38153997

[49] D. Pei , S. Yu , P. Liu , et al., “Reversible Wet‐Adhesive and Self‐Healing Conductive Composite Elastomer of Liquid Metal,” Advanced Functional Materials 32, no. 35 (2022): 2204257, 10.1002/adfm.202204257.

[50] Z. Wang , X. Xia , M. Zhu , et al., “Rational Assembly of Liquid Metal/Elastomer Lattice Conductors for High‐Performance and Strain‐Invariant Stretchable Electronics,” Advanced Functional Materials 32, no. 10 (2022): 2108336, 10.1002/adfm.202108336.

[51] Z. Li , Y.‐L. Zhu , W. Niu , et al., “Healable and Recyclable Elastomers With Record‐High Mechanical Robustness, Unprecedented Crack Tolerance, and Superhigh Elastic Restorability,” Advanced Materials 33, no. 27 (2021): 2101498, 10.1002/adma.202101498. 34062022

[52] A. R. Jacob , D. P. Parekh , M. D. Dickey , and L. C. Hsiao , “Interfacial Rheology of Gallium‐Based Liquid Metals,” Langmuir 35, no. 36 (2019): 11774–11783, 10.1021/acs.langmuir.9b01821. 31407902

[53] J. Shim , Y. U. Kim , Y.‐B. Kim , et al., “A Surface Conformal Laser‐Assisted Alloying Reaction for 3D‐Printable Solid/Liquid Biphasic Conductors,” Small Science 3, no. 3 (2023): 2200089, 10.1002/smsc.202200089. PMC11935982 40212058

[54] Q. Zhuang , Z. Ma , Y. Gao , et al., “Liquid–Metal‐Superlyophilic and Conductivity–Strain‐Enhancing Scaffold for Permeable Superelastic Conductors,” Advanced Functional Materials 31, no. 47 (2021): 2105587, 10.1002/adfm.202105587.

[55] S.‐B. Shin , G. H. Kim , J.‐H. Cho , et al., “Rational Nanoarchitecturing of Coinage Metals via Kinetically Controlled Mass‐Limited Reaction With EGaIn Nanoparticles,” Journal of the American Chemical Society 147, no. 51 (2025): 46941–46952, 10.1021/jacs.5c10684. 41391152

[56] T. Daeneke , K. Khoshmanesh , N. Mahmood , et al., “Liquid Metals: Fundamentals and Applications in Chemistry,” Chemical Society Reviews 47, no. 11 (2018): 4073–4111, 10.1039/c7cs00043j. 29611563

[57] L. Pan , F. Wang , Y. Cheng , et al., “A Supertough Electro‐Tendon Based on Spider Silk Composites,” Nature Communications 11, no. 1 (2020): 1332, 10.1038/s41467-020-14988-5. PMC7067870 32165612

[58] P. A. Lopes , D. F. Fernandes , A. F. Silva , et al., “Bi‐Phasic Ag–In–Ga‐Embedded Elastomer Inks for Digitally Printed, Ultra‐Stretchable, Multi‐Layer Electronics,” ACS Applied Materials & Interfaces 13, no. 12 (2021): 14552–14561, 10.1021/acsami.0c22206. 33689286

[59] J. E. Park , H. S. Kang , M. Koo , and C. Park , “Autonomous Surface Reconciliation of a Liquid‐Metal Conductor Micropatterned on a Deformable Hydrogel,” Advanced Materials 32, no. 37 (2020): 2002178, 10.1002/adma.202002178. 32743939

[60] W. Jung , M. H. Vong , K. Kwon , et al., “Giant Decrease in Interfacial Energy of Liquid Metals by Native Oxides,” Advanced Materials 36, no. 48 (2024): 2406783, 10.1002/adma.202406783. PMC11602690 39388528

[61] Y. Zhang , Z. Wang , S. Wang , Z. Yu , Z. Xu , and L. Jiang , “Dynamic‐Wetting Liquid Metal Thin Layer Induced via Surface Oxygen‐Containing Functional Groups,” ACS Nano 19, no. 4 (2025): 4913–4923, 10.1021/acsnano.4c16623. 39841619

[62] Y. Ding , M. Zeng , and L. Fu , “Surface Chemistry of Gallium‐Based Liquid Metals,” Matter 3, no. 5 (2020): 1477–1506, 10.1016/j.matt.2020.08.012.

[63] D. Wang , J. Ye , Y. Bai , et al., “Liquid Metal Combinatorics Toward Materials Discovery,” Advanced Materials 35, no. 52 (2023): 2303533, 10.1002/adma.202303533. 37417920

[64] X. Chen , J. Sun , J. Liang , R. Xing , and J. Kong , “Surface and Interface Engineering in Gallium‐Based Liquid Metals,” Advanced Functional Materials 36, no. 19 (2026): 19697, 10.1002/adfm.202519697.

[65] H. Jiang , B. Yuan , H. Guo , et al., “Malleable, Printable, Bondable, and Highly Conductive MXene/Liquid Metal Plasticine With Improved Wettability,” Nature Communications 15, no. 1 (2024): 6138, 10.1038/s41467-024-50541-4. PMC11271265 39033166

[66] Y. Wang , W. Qin , M. Yang , et al., “High Linearity, Low Hysteresis Ti_3_C_2_T_x_MXene/AgNW/Liquid Metal Self‐Healing Strain Sensor Modulated by Dynamic Disulfide and Hydrogen Bonds,” Advanced Functional Materials 33, no. 37 (2023): 2301587, 10.1002/adfm.202301587.

[67] L. Wu , P. Li , X. Xiao , et al., “A Programmable Island‐Bridge Configuration for Constructing Ultra‐Stretchable Conductors,” Advanced Functional Materials 35, no. 35 (2025): 2506492, 10.1002/adfm.202506492.

[68] Y. Liu , X. Zhou , P. Min , B. Shen , Z.‐Z. Yu , and H.‐B. Zhang , “Machine Learning‐Accelerated Discovery of High‐Performance Insulating Electromagnetic Interference Shielding Composites for Integrated Electronics,” Advanced Materials 38, no. 9 (2026): 18581, 10.1002/adma.202518581. 41358524

[69] X. Zhou , P. Min , Y. Liu , M. Jin , Z.‐Z. Yu , and H.‐B. Zhang , “Insulating Electromagnetic‐Shielding Silicone Compound Enables Direct Potting Electronics,” Science 385, no. 6714 (2024): 1205–1210, 10.1126/science.adp6581. 39265019

[70] G. Yin , J. Wu , C. Qi , X. Zhou , Z.‐Z. Yu , and H.‐B. Zhang , “Pickering Emulsion‐Driven MXene/Silk Fibroin Hydrogels With Programmable Functional Networks for EMI Shielding and Solar Evaporation,” Nano‐Micro Letters 17, no. 1 (2025): 312, 10.1007/s40820-025-01818-w. PMC12185816 40551046

[71] L. Sanchez‐Botero , D. S. Shah , and R. Kramer‐Bottiglio , “Are Liquid Metals Bulk Conductors?,” Advanced Materials 34, no. 26 (2022): 2109427, 10.1002/adma.202109427. 35293649

[72] W. Niu , Z. Li , F. Liang , H. Zhang , and X. Liu , “Ultrastable, Superrobust, and Recyclable Supramolecular Polymer Networks,” Angewandte Chemie International Edition 63, no. 10 (2024): 202318434, 10.1002/anie.202318434. 38234012

[73] A. N. Keith , M. Vatankhah‐Varnosfaderani , et al., “Bottlebrush Bridge Between Soft Gels and Firm Tissues,” ACS Central Science 6, no. 3 (2020): 413–419, 10.1021/acscentsci.9b01216. PMC7099586 32232141

[74] W. Yang , V. R. Sherman , B. Gludovatz , et al., “On the Tear Resistance of Skin,” Nature Communications 6, no. 1 (2015): 6649, 10.1038/ncomms7649. PMC4389263 25812485

[75] X. Zhao , X. Chen , H. Yuk , S. Lin , X. Liu , and G. Parada , “Soft Materials by Design: Unconventional Polymer Networks Give Extreme Properties,” Chemical Reviews 121, no. 8 (2021): 4309–4372, 10.1021/acs.chemrev.0c01088. PMC9217625 33844906

[76] M. Li , L. Chen , Y. Li , et al., “Superstretchable, Yet Stiff, Fatigue‐Resistant Ligament‐Like Elastomers,” Nature Communications 13, no. 1 (2022): 2279, 10.1038/s41467-022-30021-3. PMC9046184 35477583

[77] J. Kim , G. Zhang , M. Shi , and Z. Suo , “Fracture, Fatigue, and Friction of Polymers in which Entanglements Greatly Outnumber Cross‐Links,” Science 374, no. 6564 (2021): 212–216, 10.1126/science.abg6320. 34618571

[78] G. J. Lake and A. G. Thomas , “The Strength of Highly Elastic Materials,” Proceedings of the Royal Society of London Series A Mathematical and Physical Sciences 300, no. 1460 (1967): 108–119, 10.1098/rspa.1967.0160.

[79] C. Li , H. Yang , Z. Suo , and J. Tang , “Fatigue‐Resistant Elastomers,” Journal of the Mechanics and Physics of Solids 134 (2020): 103751, 10.1016/j.jmps.2019.103751.

[80] N. Kazem , M. D. Bartlett , and C. Majidi , “Extreme Toughening of Soft Materials With Liquid Metal,” Advanced Materials 30, no. 22 (2018): 1706594, 10.1002/adma.201706594. 29663540

[81] F. Sun , L. Liu , T. Liu , et al., “Vascular Smooth Muscle‐Inspired Architecture Enables Soft Yet Tough Self‐Healing Materials for Durable Capacitive Strain‐Sensor,” Nature Communications 14, no. 1 (2023): 130, 10.1038/s41467-023-35810-y. PMC9829674 36624140

[82] C. Cao , X. Huang , D. Lv , et al., “Ultrastretchable Conductive Liquid Metal Composites Enabled by Adaptive Interfacial Polarization,” Materials Horizons 8, no. 12 (2021): 3399–3408, 10.1039/d1mh00924a. 34679157

[83] S. Chen , S. Fan , J. Qi , et al., “Ultrahigh Strain‐Insensitive Integrated Hybrid Electronics Using Highly Stretchable Bilayer Liquid Metal Based Conductor,” Advanced Materials 35, no. 5 (2023): 2208569, 10.1002/adma.202208569. 36353902

[84] S. Liu , D. S. Shah , and R. Kramer‐Bottiglio , “Highly Stretchable Multilayer Electronic Circuits Using Biphasic Gallium‐Indium,” Nature Materials 20, no. 6 (2021): 851–858, 10.1038/s41563-021-00921-8. 33603186

[85] B. Ma , C. Xu , J. Chi , J. Chen , C. Zhao , and H. Liu , “A Versatile Approach for Direct Patterning of Liquid Metal Using Magnetic Field,” Advanced Functional Materials 29, no. 28 (2019): 1901370, 10.1002/adfm.201901370.

[86] C. J. Thrasher , Z. J. Farrell , N. J. Morris , C. L. Willey , and C. E. Tabor , “Mechanoresponsive Polymerized Liquid Metal Networks,” Advanced Materials 31, no. 40 (2019): 1903864, 10.1002/adma.201903864. 31403234

[87] L. Yang , Z. Wang , H. Wang , et al., “Self‐Healing, Reconfigurable, Thermal‐Switching, Transformative Electronics for Health Monitoring,” Advanced Materials 35, no. 15 (2023): 2207742, 10.1002/adma.202207742. PMC10391699 36719993

[88] L. Zheng , M. Zhu , B. Wu , Z. Li , S. Sun , and P. Wu , “Conductance‐Stable Liquid Metal Sheath‐Core Microfibers for Stretchy Smart Fabrics and Self‐Powered Sensing,” Science Advances 7, no. 22 (2021): abg4041, 10.1126/sciadv.abg4041. PMC8163087 34049879

[89] Y. Xu , Y. Su , X. Xu , et al., “Porous Liquid Metal–Elastomer Composites With High Leakage Resistance And Antimicrobial Property For Skin‐Interfaced Bioelectronics,” Science Advances 9, no. 1 (2023): adf0575, 10.1126/sciadv.adf0575. PMC9821854 36608138

[90] H. Ye , B. Wu , S. Sun , and P. Wu , “A Solid–Liquid Bicontinuous Fiber With Strain‐Insensitive Ionic Conduction,” Advanced Materials 36, no. 25 (2024): 2402501, 10.1002/adma.202402501. 38562038

[91] B. Tian , Y. Wang , N. Jiang , Z. Chen , and J. Zhang , “Chemical Exchange Between Silica Networks and Liquid Metals for All‐Inorganic, Sintering‐Free and Highly Conductive Inks,” Advanced Materials 37, no. 37 (2025): 2501414, 10.1002/adma.202501414. 40536200

[92] W. Zhao , Y. Deng , B. Deng , et al., “Modulus Engineered Substrate With Vertical Soft Interconnects for Ultra‐Stable Stretchable Multilayer Electronic Systems,” Advanced Materials 38, no. 13 (2026): 72292, 10.1002/adma.72292. 41588877

[93] J. Wang , M.‐F. Lin , S. Park , and P. S. Lee , “Deformable Conductors For Human–Machine Interface,” Materials Today 21, no. 5 (2018): 508–526, 10.1016/j.mattod.2017.12.006.

[94] W. Wang , J. Yang , B. Song , et al., “Advanced Liquid Metal Interfaces: Engineering Embodied Cognition in Closed‐Loop Human‐Machine Ecosystems,” Science Bulletin 71, no. 6 (2026): 1498–1523, 10.1016/j.scib.2026.01.073. 41708381

[95] S. Ahmed , M. Momin , J. Ren , H. Lee , and T. Zhou , “Self‐Assembly Enabled Printable Asymmetric Self‐Insulated Stretchable Conductor for Human Interface,” Advanced Materials 36, no. 25 (2024): 2400082, 10.1002/adma.202400082. 38563579

[96] Y. Jung , M. Kim , S. Jeong , S. Hong , and S. H. Ko , “Strain‐Insensitive Outdoor Wearable Electronics by Thermally Robust Nanofibrous Radiative Cooler,” ACS Nano 18, no. 3 (2024): 2312–2324, 10.1021/acsnano.3c10241. 38190550

[97] W. Niu , Q. Tian , Z. Liu , and X. Liu , “Solvent‐Free and Skin‐Like Supramolecular Ion‐Conductive Elastomers With Versatile Processability for Multifunctional Ionic Tattoos and On‐Skin Bioelectronics,” Advanced Materials 35, no. 39 (2023): 2304157, 10.1002/adma.202304157. 37345560

