# Supplementary material for: A Superstrong and Ultratough Stretchable Electronic Conductor With Ultradurable Strain‐Insensitive Electromechanical Performance
Source: Adv Sci (Weinh). 2026 Sep 27:e77457. Online ahead of print. doi: 10.1002/advs.77457 (PMC13616252; doi:10.1002/advs.77457)
Supplement: Supplementary file 1 [file ADVS-9999-e77457-s005.docx]

Supporting Information for

A Superstrong and Ultratough Stretchable Electronic Conductor with Ultradurable Strain-Insensitive Electromechanical Performance

*Yuxing Shan, Dong Lei, Chengzhi Huang, Chunhong Gong, Jingwei Zhang, and Xiaokong Liu**

**Table of Contents**

**1. Experimental Section**	S3

1.1. Materials	S3

1.2. Synthesis of the SPU	S3

1.3. Synthesis of the EGaInAg alloys	S3

1.4. Synthesis of EGaInAg-SPU	S4

1.5. Synthesis of EGaIn-SPU-Ag	S4

1.6. Synthesis of EGaIn-SPU	S4

1.7. Synthesis of EGaInAg-SEBS	S4

1.8. Characterization	S4

1.9. Electrical measurement and characterization	S5

1.10. Tensile tests	S6

1.11. Measurement of the fatigue threshold	S6

1.12. Signal transmission test	S7

1.13. Recording of ECG signals	S7

1.14. Recording of EMG signals	S7

**2. Supplementary Figures and Tables**	S9

**3. Supplementary Movies**	S29

**4. References**	S29

**1. Experimental Section**

**1.1. Materials**

Eutectic gallium-indium alloy (EGaIn LM, 75.5 wt% Ga, 24.5 wt% In, melting point 15.7 °C) was purchased from Jia Chun Co., Ltd. Silver (Ag) microflakes (diameter: 5-10 μm, 99.99%), adipic dihydrazide (AD, 98%), and dibutyltin dilaurate (DBTDL, 95%) were purchased from Sigma-Aldrich. Polytetramethylene ether glycol (PTMEG, Mn = 2000 g mol^-1^) and isophorone diisocyanate (IPDI, 99%) were purchased from Aladdin (China). N,N-dimethylacetamide (DMAc, 99.8%) was purchased from Beijing InnoChem Science & Technology Co., Ltd. Toluene (99.5%) was purchased from Tianjin Fuyu Fine Chemical Co., Ltd. Polystyrene-block-poly(ethylene-ran-butylene)-block-polystyrene (SEBS, Product: H1221) was purchased from Asahi Kasei. All reagents and solvents were used as received without further purification.

**1.2. Synthesis of the SPU**

SPU polymer was synthesized according to our previously reported method.^[1]^ A three-neck round bottom flask equipped with a mechanical stirrer was charged with PTMEG (*M*n of 2000 g mol^-1^, 10.00 g), followed by heating at 110 ℃ under vacuum and stirring for 30 min to remove the moisture. After cooling the reaction system to 80 ℃, the mixture of IPDI (2.22 g, 10 mmol) and dibutyltin dilaurate (DBTDL, 0.02 g) dissolved in N,N-dimethylacetamide (DMAc, 10 mL) was added into the flask. The reaction system was further heated at 80 ℃ under N_2_ atmosphere and stirring for 3 h, followed by being cooled to 40 ℃. Consequently, AD (0.87 g, 5 mmol) as the chain extender, dissolved in DMAc (60 mL), was added into the reaction system that was further kept at 40 ℃ for 15 h under stirring and N_2_ atmosphere. Viscous and transparent DMAc solution of the SPU polymer was finally obtained.

**1.3. Synthesis of the** **EGaInAg alloys**

EGaInAg alloys were prepared by grinding Ag microflakes into EGaIn LM for 10 min until a homogeneous viscoplastic paste without visible Ag flakes or phase separation was obtained. The weight fraction of Ag microflakes was varied from 5%, 9%, and 17%.

**1.4. Synthesis of** **EGaInAg-SPU**

EGaInAg_9%_ alloy (2.75 g) was dispersed into 3.00 g DMAc solution of SPU (17 wt%) under high-speed stirring (10,000 rpm) for 10 min. The resultant suspension was cast into a petri dish (6.5 cm in diameter), heated on a hot plate at 80 °C overnight, and dried at 80 °C for 48 h under vacuum to obtain the composite sample.

**1.5. Synthesis of** **EGaIn-SPU-Ag**

EGaIn LM (2.50 g) and Ag microflakes (0.25 g) were separately dispersed into 3.00 g DMAc solution of SPU (17 wt%) under high-speed stirring (10,000 rpm) for 10 min. The resultant suspension was cast into a petri dish (6.5 cm in diameter), heated on a hot plate at 80 °C overnight, and dried at 80 °C for 48 h under vacuum to obtain the composite sample.

**1.6. Synthesis of EGaIn-SPU**

EGaIn LM (2.75 g) was dispersed into 3.00 g DMAc solution of SPU (17 wt%) under high-speed stirring (10,000 rpm) for 10 min. The resultant suspension was cast into a petri dish (6.5 cm in diameter), heated on a hot plate at 80 °C overnight, and dried at 80 °C for 48 h under vacuum to obtain the composite sample.

**1.7. Synthesis of** **EGaInAg-SEBS**

EGaInAg_9%_ alloy (2.75 g) was dispersed into 3.00 g toluene solution of SEBS (17 wt%) under high-speed stirring (10,000 rpm) for 10 min. The resulting suspension was cast into a petri dish (6.5 cm in diameter), heated at 80 °C overnight, and vacuum-dried at 80 °C for 48 h to obtain the composite sample.

**1.8. Characterization**

Scanning electron microscopy (SEM) images were obtained on the Hitachi Regulus 8100 operated at 20 kV under vacuum. In order to see the internal structure of EGaInAg-SPU without inducing any damage from cutting, the sample was cryogenically treated by immersing it in liquid nitrogen for 1 min to induce low-temperature brittleness. Subsequently, the sample was promptly removed and bent immediately to break it into two pieces, after which the newly formed fracture surface was subjected to imaging. Energy-dispersive X-ray spectroscopy (EDS) analyses were performed with a Hitachi Regulus 8100 scanning electron microscope, equipped with a Bruker EDS system. To prepare samples for X-ray photoelectron spectroscopy (XPS) analysis of internal gallium species, EGaIn LM and EGaInAg alloys were transferred into a nitrogen glovebox to prevent oxidation. Fresh internal surfaces were exposed by slicing the samples with a scalpel under inert conditions, and the materials were immediately mounted for measurement. High-resolution XPS spectra were acquired using a PREVAC R3000 spectrometer with a monochromatic Al Kα X-ray source (1486.7 eV). X-ray diffraction (XRD) measurements were conducted on a Rigaku SmartLab X-Ray diffractometer using Cu Kα radiation with the 2θ range of 10°-90° and collected with a step-width of 0.02°, at the accelerating voltage of 40 kV and emission current of 30 mA. Rheological measurements were performed with a Waters Corporation DHR-2 at 25 ℃. Optical microscopy images were recorded using an Olympus DSX1000 digital microscope. Digital photographs were taken using a Sony Alpha 7 III camera.

**1.9. Electrical measurement and characterization.**

Electrical conductivity measurement: The samples were cut into stripes by a cutting machine (30 mm × 6 mm). The thickness of the EGaInAg-rich layer was determined using SEM. To minimize contact resistance, bulk EGaIn LM droplets were applied to both ends of each strip to serve as contact interfaces with the measurement probes. The resistance of the samples was measured using the four-terminal method with equally spaced probes connected to a Keysight 34465A 6_1/2_-digit multimeter. The initial conductivity was then calculated by the following equation:

$\sigma=\frac{L}{R*S}$	(S1)

where L, R, and S represent the distance between the electrodes, the measured resistance, and the cross-sectional area of the conductive layer, respectively.

For electromechanical characterization, the resistance of the samples under different strain states was measured using a Keysight 34465A 6_1/2_-digit multimeter in conjunction with a computer-controlled stretching motor. The sample was cut into a small strip with dimensions of 30 mm × 6 mm and mounted on the motion platform. The computer-controlled motor then stretched the strip to various lengths. The four-terminal method was employed to measure the resistance changes during stretching, eliminating the influence of contact resistance. [The connecting parts to the sample were at the two ends of the strip, which were fixed with highly conductive copper tape to ensure good contact](https://paperpal.com/).

**1.10. Tensile tests.**

Tensile tests were performed at room temperature using a universal testing machine (Instron 65TM-5, 5 kN) at a stretching speed of 50 mm min⁻^1^. Dumbbell-shaped samples were prepared with an effective gauge dimension of 14 mm length, 2 mm width, and 0.2 mm thickness. At least five individual samples were tested, and the average values were reported. True stress ($\sigma_{t}$) is calculated based on the engineering stress-strain curves, by the following equation:

$\sigma_{t}=\sigma*\frac{L}{L_{0}}=\sigma*(\varepsilon+1)$	(S2)

where 𝜎 is the engineering stress, 𝐿 is the instant length of the deformed specimen, 𝐿_0_ is the original length of the specimen, 𝜀 is the engineering strain.

**1.11. Measurement of the fatigue threshold.**

The fatigue resistance of the EGaInAg-SPU SEC was evaluated by cyclic tensile tests on the pre-notched specimens. All fatigue tests were conducted in air at 25  ±  1 °C using a universal testing machine (Instron 65TM-5) at a tensile loading rate of 50  mm min⁻^1^. Rectangular specimens were 5  mm wide, 0.2  mm thick, with a gauge length of 10  mm and an initial crack width of 1  mm. Crack propagation was monitored using a digital camera (Alpha 7 III, Sony). Two identical specimens (one notched, one unnotched) were loaded under the specimen setup as a pair. The notched specimens were used to obtain the $\lambda^{A}$ and the stress-strain curves of the unnotched specimens were used to calculate the energy release rate value *G*.

$G\left( \lambda^{A},N \right)=2k\left( \lambda^{A} \right)*c(N)*W(\lambda^{A},N)$	(S3)

where *k* is a function of strain variation determined by $k=3/\sqrt{\lambda^{A}}$, *c* is the crack length measured in the undeformed configuration, and *W* is the strain energy density of unnotched specimens of the same dimensions stretched to the same cyclic stretch $\lambda^{A}$. The strain energy density *W* of the unnotched sample under the *N*th cycle can be obtained using:

$W(\lambda^{A},N)=\int_{1}^{\lambda^{A}} \sigma d\lambda$	(S4)

**1.12. Signal transmission test.**

Various alternating voltage signals were generated from a signal generator (JDS6600, HQXRTEK) and transmitted through two EGaInAg-SPU SECs (~30 mm × 2.0 mm × 0.2 mm) to a signal acquisition system (DS1054, RIGOL).

**1.13. Recording of ECG signals.**

ECG signals were recorded using a multi-modal signal acquisition and analysis system with three electrodes. Two electrodes were attached to the forearms, and one reference electrode was placed on the abdomen. All electrodes were connected to the signal acquisition unit via EGaInAg-SPU SECs.

**1.14. Recording of EMG signals.**

EMG signals were recorded using a multi-modal signal acquisition and analysis system with two sets of electrodes connected via EGaInAg-SPU SECs. Reference electrodes were placed on the wrist, while monitoring electrodes were attached to the forearm.

Raw data were sampled at 2 kHz and bandpass filtered between 20 and 500 Hz. Power-line interference at 50 Hz and its harmonics was also filtered out. The signal-to-noise ratio (SNR) was calculated as follows:

$SNR(dB)=20log₁₀ \frac{A_{signal}}{A_{noise}}$	(S5)

where 𝐴_𝑠𝑖𝑔𝑛𝑎𝑙_ is the mean absolute value of each EMG waveform, and 𝐴_𝑛𝑜𝑖𝑠𝑒_ is the mean absolute value of a 0.1 s segment shifted 0.2 s earlier relative to the signal window.

**2. Supplementary Figures and Tables**

**Figure S1.** Preparation of EGaInAg alloy.

**Figure S2.** Photographs of EGaIn LM and EGaInAg alloys.

**Figure S3.** SEM images of Ag microflakes, EGaIn LM, and EGaInAg_9%_ alloy.

**Figure S4.** Viscosity of EGaIn and EGaInAg alloys. Plot of apparent steady-state viscosity as a function of shear rate. Data were collected at room temperature (25 ℃).

**Figure S5.** a) Photograph and magnified image of resistance measurement using a digital multimeter with the four-terminal method. Scale bar, 1 cm. b) The equivalent circuit of the four-terminal method. c) Conductivity of EGaInAg alloys with 5%, 9%, and 17% Ag microflake content.

**Figure S6.** a) High-resolution XPS spectra of Ga 2p for EGaIn LM and EGaInAg alloys. b) Relative contents of oxidized gallium species (Ga_2_O_3_ and Ga_2_O) with respect to total Ga 2p signals in EGaIn LM and EGaInAg alloys.

**Figure S7.** Schematic synthesis route of SPU.

**Figure S8.** Preparation of the EGaInAg-SPU composite.

**Figure S9.** a-c) Schematic illustrations of the preparation processes (top) and photographs of the leakage behavior (bottom) of EGaInAg-SPU (a), EGaIn-SPU-Ag (b), and EGaIn-SPU (c) films during peeling from petri dishes.

In sharp contrast to the (EGaInAg_9%_)_85%_-SPU SEC that exhibits no LM leakage during the peeling process, both the EGaIn-SPU-Ag and EGaIn-SPU samples display substantial LM leakage, highlighting that the quasi-solid EGaInAg_9%_ alloy plays a critical role in preventing LM leakage.

**Figure S10.** Size distribution of EGaInAg_9%_ agglomerates in EGaInAg-SPU.

**Figure S11.** a,b) SEM images and corresponding EDS mapping images of the cross-section of EGaInAg-SPU at low magnification (a) and high magnification (b).

**Figure S12.** XRD patterns of EGaInAg-SPU and EGaIn-SPU-Ag. Standard reference patterns for AgIn_2_ (PDF#00-025-0386), In_4_Ag_9_ (PDF#00-029-0678), and Ag (PDF#00-004-0783) are included for comparison.

**Figure S13.** a,b) SEM images and corresponding EDS mapping images of the cross-section of EGaIn-SPU-Ag at low magnification (a) and high magnification (b).

**Figure S14.** Photographs of the cyclic adhesive-tape-tapping setup used to evaluate LM leakage in LM-based SECs.

**Figure S15.** *R/R*_0_ of EGaIn*_y%_*-SPU and (EGaInAg_9%_)*_y%_*-SPU during adhesive-tape-tapping tests.

**Figure S16.** EDS elemental maps of the LM-rich surface of EGaInAg-SPU in the pristine state (top), under 300% and 500% strain (middle), and after recovery (bottom). Scale bar, 100 μm.

**Table S1.** Comparison of Young’s modulus, tensile strength, elongation-at-break, and toughness of the EGaInAg-SPU with state-of-the-art LM-based SECs.

| **Sample** | **Young’s modulus**  **(MPa)** | **Tensile strength (MPa)** | **Elongation-at-break (%)** | **Toughness**  **(MJ m^-3^)** | **Ref.** |
| --- | --- | --- | --- | --- | --- |
| **EGaInAg-SPU** | **3.90** | **20.01** | **1023** | **66.30** | **This work** |
| EGaIn/PU | 56.70 | 9.23 | 560 | 27.95 | ^[2]^ |
| EGaIn/Sylgard184 | 3.49 | 1.13 | 200 | 1.61 |  |
| EGaIn/PVDF-HFP | 8.49 | 0.67 | 4100 | 0.43 |  |
| EGaIn/Self-healing polymer | 38.50 | 1.87 | 410 | 4.80 |  |
| EGaIn/SBS | 0.20 | 0.68 | 2040 | 8.67 | ^[3]^ |
| EGaIn/SBS/PAAND | 0.85 | 0.90 | 900 | 3.58 | ^[4]^ |
| EGaIn/UPy-PDMS-NH_2_ | 10.30 | 3.20 | 965 | 19.80 | ^[5]^ |
| EGaIn/SIS/PBD | 0.44 | 0.76 | 1200 | 4.76 | ^[6]^ |
| EGaIn/PDMS | 1.27 | 3.44 | 133 | 1.61 | ^[7]^ |
| EGaIn/AgNW/PBU-COOH | 0.10 | 0.05 | 2100 | 1.13 | ^[8]^ |
| EGaIn/[TA]:[TA-DPA]:[TA-C_12_] | 10.00 | 1.36 | 52 | 0.50 | ^[9]^ |
| EGaIn/PDMS | 6.50 | 2.63 | 186 | 2.93 | ^[10]^ |
| EGaIn/PU | 0.26 | 2.57 | 1100 | 10.07 | ^[11]^ |

**Figure S17.** True stress-strain curve of EGaInAg-SPU.

**Figure S18.** Fatigue resistance of EGaInAg-SPU. Images of pre-notched EGaInAg-SPU samples (5-mm width with 1-mm notch) stretched to different stretch ratios (λ = 1.5-2.0), captured after 1, 5,000, and 10,000 loading cycles at each λ. The stress-strain curves of the unnotched EGaInAg-SPU after 10,000 cycles are also shown. Here, *G*(*λ*) represents the energy release rate value. Scale bar, 1 mm.

**Figure S19.** Longitudinal crack propagation in EGaInAg-SPU under 200% strain.

**Figure S20.** SEM images of the LM-rich surfaces of EGaInAg-SPU, EGaIn-SPU, EGaIn-SPU-Ag, and EGaInAg-SEBS in their pristine state (top) and after 1,000 cycles of tensile loading at 300% strain (bottom). Scale bar, 500 μm. Red dashed circles highlight leakage of EGaIn LM or EGaInAg, while yellow dashed circles indicate cracks.

**Figure S21.** a,b) The structural and chemical stability of the conductive pathways in the EGaInAg-SPU (a) and EGaIn-SPU-Ag (b) SECs were investigated by EDS mapping (left) and high-resolution XPS spectra of Ga 2p (right) in the pristine state and after 1,000 cycles of tensile loading at 300% strain. Scale bar, 10 μm.

In the EGaInAg-SPU SEC, EDS mapping and XPS analyses show that the EGaInAg agglomerates and the oxidation state of Ga remain unchanged after 1,000 stretching cycles (a), indicating both structural and chemical stability under prolonged cyclic deformation. In contrast, the EGaIn microdroplets in the EGaIn-SPU-Ag SEC exhibit progressive wrinkling, indicating LM leakage and oxidation, as evidenced by the fully oxidized Ga 2p XPS spectrum (b).

**Table S2** Comparison of the strain sensitivity and long-term cyclic electromechanical stability at 100% strain between EGaInAg-SPU and previously reported LM-based SECs.

| **Sample** | ***R/R*_0_**  **at 100% strain** | **Stretching-releasing cycles at 100% strain** | **Resistance change** | **Ref.** |
| --- | --- | --- | --- | --- |
| **EGaInAg-SPU** | **1.01** | **20,000** | **1%** | **This work** |
| EGaIn/PU | 1.00 | 15,000 | 8% | ^[2]^ |
| EGaIn/SEBS | 1.00 | 1,500 | 11% |  |
| bGaIn | 1.16 | 5,000 | 4% | ^[12]^ |
| bGaIn/PDMS | 1.06 | 1,500 | 9% | ^[13]^ |
| EGaIn/SBS | 1.00 | 20,000 | 26% | ^[3]^ |
| EGaIn/SBS/PAAND | 1.43 | 10,000 | 33% | ^[4]^ |
| bGaIn | 1.25 | 2,000 | 11% | ^[14]^ |
| EGaIn/pp-TPU | 1.06 | 10,000 | 5% | ^[15]^ |
| EGaIn/PVDF-co-CTFE | 1.13 | 4,500 | 2% | ^[16]^ |
| EGaIn/SIS/PBD | / | 1,000 | 46% | ^[6]^ |
| Poly-EGaIn | 1.07 | 10,000 | 1% | ^[17]^ |
| EGaIn-Ag-SIS | / | 1,000 | 50% | ^[18]^ |
| PVDF-HFP-TFE-LM | 3.1 | 600 | 17% | ^[19]^ |
| Ni-EGaIn | 1.46 | 2,000 | 2% | ^[20]^ |

**Table S3** Comparison of the strain sensitivity and long-term cyclic electromechanical stability at 500% strain between EGaInAg-SPU and previously reported LM-based SECs.

| **Sample** | ***R/R*_0_**  **at 500% strain** | **Stretching-releasing cycles at 500% strain** | **Resistance change (%)** | **Ref.** |
| --- | --- | --- | --- | --- |
| EGaInAg-SPU | 1.15 | 8,000 | 15% | This work |
| EGaIn/PU | 1.13 | 1,200 | 22% | ^[2]^ |
| EGaIn/PU | 1.30 | 1,000 | 15% | ^[21]^ |
| bGaIn/VHB | 1.81 | 1,500 | 38% | ^[13]^ |
| bGaIn | 1.64 | 5,000 | 29% | ^[12]^ |
| EGaIn/SBS | 1.00 | 1,000 | 21% | ^[3]^ |
| EGaIn/PVDF-co-CTFE | 1.44 | 6,000 | 225% | ^[16]^ |

**3. Supplementary Movies**

**Movie S1.** Interfacial adhesion of EGaInAg_9%_ alloy and EGaIn LM droplet on SPU elastomer under an Ar atmosphere

**Movie S2.** High-fidelity electrical signal transmission through EGaInAg-SPU SECs during dynamic stretching

**Movie S3.** Wearable electronic system based on EGaInAg-SPU SECs for high-fidelity ECG signal recording during repeated elbow extension and bending

**Movie S4.** Wearable electronic system based on EGaInAg-SPU SECs for reliable EMG recording during grip-force application under both elbow extension and flexion

**Movie S5.** Wearable human-machine interface based on EGaInAg-SPU SECs for real-time robotic hand control under dynamic deformation

4. References

1.	Z. Li, Y. L. Zhu, W. Niu, et al., “Healable and Recyclable Elastomers with Record-High Mechanical Robustness, Unprecedented Crack Tolerance, and Superhigh Elastic Restorability,” *Advanced Materials* 33, no. 27 (2021): 2101498. <https://doi.org/10.1002/adma.202101498>

2.	W. Lee, H. Kim, I. Kang, et al., “Universal Assembly of Liquid Metal Particles in Polymers Enables Elastic Printed Circuit Board,” *Science* 378, no. 6620 (2022): 637–641. <https://doi.org/10.1126/science.abo6631>

3.	Z. Ma, Q. Huang, Q. Xu, et al., “Permeable Superelastic Liquid-Metal Fibre Mat Enables Biocompatible and Monolithic Stretchable Electronics,” *Nature Materials* 20, no. 6 (2021): 859–868. <https://doi.org/10.1038/s41563-020-00902-3>

4.	F. Chen, Q. Zhuang, Y. Ding, et al., “Wet-Adaptive Electronic Skin,” *Advanced Materials* 35, no. 49 (2023): 2305630. <https://doi.org/10.1002/adma.202305630>

5.	L. Ai, W. Lin, C. Cao, et al., “Tough Soldering for Stretchable Electronics by Small-Molecule Modulated Interfacial Assemblies,” *Nature Communications* 14, no. 1 (2023): 7723. <https://doi.org/10.1038/s41467-023-43574-8>

6.	R. Tutika, A. B. M. T. Haque, and M. D. Bartlett, “Self-Healing Liquid Metal Composite for Reconfigurable and Recyclable Soft Electronics,” *Communications Materials* 2, no. 1 (2021): 64. <https://doi.org/10.1038/s43246-021-00169-4>

7.	A. Fassler, and C. Majidi, “Liquid-Phase Metal Inclusions for a Conductive Polymer Composite,” *Advanced Materials* 27, no. 11 (2015): 1928–1932. <https://doi.org/10.1002/adma.201405256>

8.	W. Zang, Y. Wang, W. Wu, et al., “Superstretchable Liquid-Metal Electrodes for Dielectric Elastomer Transducers and Flexible Circuits,” *ACS Nano* 18, no. 1 (2024): 1226–1236. <https://doi.org/10.1021/acsnano.3c12210>

9.	D. Pei, S. Yu, P. Liu, et al., “Reversible Wet-Adhesive and Self-Healing Conductive Composite Elastomer of Liquid Metal,” *Advanced Functional Materials* 32, no. 35 (2022): 2204257. <https://doi.org/10.1002/adfm.202204257>

10.	Z. Wang, X. Xia, M. Zhu, et al., “Rational Assembly of Liquid Metal/Elastomer Lattice Conductors for High-Performance and Strain-Invariant Stretchable Electronics,” *Advanced Functional Materials* 32, no. 10 (2022): 2108336. <https://doi.org/10.1002/adfm.202108336>

11.	X. Li, J. Wang, W. Wang, et al., “A Durable Metalgel Maintaining 3×10^6^ S∙m^‒1^ Conductivity Under 1 000 000 Stretching Cycles,” *Advanced Materials* 37, no. 20 (2025): 2420628. <https://doi.org/10.1002/adma.202420628>

12.	D. H. Lee, T. Lim, J. Pyeon, et al., “Self-Mixed Biphasic Liquid Metal Composite with Ultra-High Stretchability and Strain-Insensitivity for Neuromorphic Circuits,” *Advanced Materials* 36, no. 16 (2024): 2310956. <https://doi.org/10.1002/adma.202310956>

13.	S. Liu, D. S. Shah, and R. Kramer-Bottiglio, “Highly Stretchable Multilayer Electronic Circuits Using Biphasic Gallium-Indium,” *Nature Materials* 20, no. 6 (2021): 851–858. <https://doi.org/10.1038/s41563-021-00921-8>

14.	L. Yang, Z. Wang, H. Wang, et al., “Self-Healing, Reconfigurable, Thermal-Switching, Transformative Electronics for Health Monitoring,” *Advanced Materials* 35, no. 15 (2023): 2207742. <https://doi.org/10.1002/adma.202207742>

15.	S. Chen, S. Fan, J. Qi, et al., “Ultrahigh Strain-Insensitive Integrated Hybrid Electronics Using Highly Stretchable Bilayer Liquid Metal Based Conductor,” *Advanced Materials* 35, no. 5 (2023): 2208569. <https://doi.org/10.1002/adma.202208569>

16.	C. Cao, X. Huang, D. Lv, et al., “Ultrastretchable Conductive Liquid Metal Composites Enabled by Adaptive Interfacial Polarization,” *Materials Horizons* 8, no. 12 (2021): 3399–3408. <https://doi.org/10.1039/d1mh00924a>

17.	C. J. Thrasher, Z. J. Farrell, N. J. Morris, C. L. Willey, and C. E. Tabor, “Mechanoresponsive Polymerized Liquid Metal Networks,” *Advanced Materials* 31, no. 40 (2019): 1903864. <https://doi.org/10.1002/adma.201903864>

18.	M. Tavakoli, P. Alhais Lopes, A. Hajalilou, et al., “3R Electronics: Scalable Fabrication of Resilient, Repairable, and Recyclable Soft-Matter Electronics,” *Advanced Materials* 34, no. 31 (2022): 2203266. <https://doi.org/10.1002/adma.202203266>

19.	L. Zheng, M. Zhu, B. Wu, Z. Li, S. Sun, and P. Wu, “Conductance-Stable Liquid Metal Sheath-Core Microfibers for Stretchy Smart Fabrics and Self-Powered Sensing,” *Science Advances* 7, no. 22 (2021): eabg4041. <https://doi.org/10.1126/sciadv.abg4041>

20.	B. Ma, C. Xu, J. Chi, J. Chen, C. Zhao, and H. Liu, “A Versatile Approach for Direct Patterning of Liquid Metal Using Magnetic Field,” *Advanced Functional Materials* 29, no. 28 (2019): 1901370. <https://doi.org/10.1002/adfm.201901370>

21.	Y. Xu, Y. Su, X. Xu, et al., “Porous Liquid Metal-Elastomer Composites with High Leakage Resistance and Antimicrobial Property for Skin-Interfaced Bioelectronics,” *Science Advances* 9, no. 1 (2023): eadf0575. <https://doi.org/10.1126/sciadv.adf0575>
